# Differential Pharmacodynamic Effects on Psoriatic Biomarkers by Guselkumab Versus Secukinumab Correlate with Long-Term Efficacy: An ECLIPSE Substudy

**DOI:** 10.1016/j.xjidi.2024.100297

**Published:** 2024-06-26

**Authors:** Andrew Blauvelt, Yanqing Chen, Patrick J. Branigan, Xuejun Liu, Samuel DePrimo, Brice E. Keyes, Monica Leung, Steven Fakharzadeh, Ya-Wen Yang, Ernesto J. Muñoz-Elías, James G. Krueger, Richard G. Langley

**Affiliations:** 1 Blauvelt Consulting LLC, Lake Oswego, Oregon, USA; 2 Janssen Research & Development, LLC, San Diego, California, USA; 3 Janssen Research & Development, LLC, Spring House, Pennsylvania, USA; 4 Janssen Pharmaceutical Companies of Johnson & Johnson, LLC, Horsham, Pennsylvania, USA; 5 Laboratory for Investigative Dermatology, The Rockefeller University, New York, New York, USA; 6 Department of Medicine, Faculty of Medicine, Dalhousie University, Halifax, Canada

**Keywords:** Guselkumab, PD, Psoriasis, Secukinumab

## Abstract

IL-23 is a cytokine produced by myeloid cells that drives the T helper 17 pathway and plays an essential role in the pathophysiology of plaque psoriasis. IL-23 activation initiates a cascade of cytokines subsequently inducing the expression of many psoriasis-related proteins. This study aimed to better understand the underlying mechanisms driving the differences between IL-23 and IL-17A blockade in patients with psoriasis and their implications for durability of clinical responses. Serum and/or skin biopsies were isolated from patients treated with guselkumab or secukinumab for evaluation of potential biomarkers of pharmacodynamic response to treatment. Guselkumab treatment led to significantly greater reductions of IL-17F and IL-22 serum levels than treatment with secukinumab at weeks 24 and 48, demonstrating sustained regulation of the IL-23/T helper 17 pathway. Analyses of proteomic and transcriptomic profiles of patient sera and skin biopsies demonstrated differential regulation of proteins involved in chemokine, TNF, and relevant immune signaling pathways to a greater degree with guselkumab than with secukinumab treatment. These data provide insights into the differences between the mechanisms and impact of IL-23 and IL-17A blockade in psoriasis, with implications for efficacy observations and treatment paradigms. Trial Registration: The original study was registered at ClinicalTrials.gov (NCT03090100).

## Introduction

Over the last decade, there has been substantial progress in identifying the underlying inflammatory mechanisms involved in the pathogenesis of psoriasis and in developing therapeutic agents that target these pathways. In particular, IL-23, a cytokine produced by myeloid cells that drives the T helper 17 (Th17) pathway and other type 17 responses, plays a central role in the pathophysiology of plaque psoriasis (Kim et al, 2022; Nussbaum et al., 2021). Th17 and Tc17 cells activated by IL-23 produce a number of cytokines, including IL-17A, IL-17F, and IL-22, which act as effector cytokines that subsequently induce the expression of many psoriasis-related proteins (Hawkes et al, 2018). The role of the IL-23/IL-17 pathway as a key driver of skin inflammation is supported by the clinical efficacy of agents that block IL-23 or effector cytokines regulated by IL-23 (eg, IL-17A alone or in combination with IL-17F) in the treatment of plaque psoriasis (Blauvelt et al, 2017a, 2017b; Langley et al, 2014; Reich et al, 2019; Sawyer et al, 2019).

Guselkumab, an anti-IL-23p19 subunit-specific mAb, achieved high clinical response rates in the treatment of moderate-to-severe plaque psoriasis and demonstrated superiority over the anti-TNF agent, adalimumab, in the VOYAGE 1 and 2 trials (Blauvelt et al, 2017b; Reich et al, 2017). Moreover, high levels of clinical response were maintained through 5 years of treatment with guselkumab in both studies (Reich et al, 2021a). When compared with secukinumab, an IL-17A inhibitor, in the ECLIPSE trial, a significantly greater proportion of patients with moderate-to-severe psoriasis treated with guselkumab achieved the primary endpoint of at least 90% improvement in PASI (PASI 90) at week 48 (84 vs 70%; *P* <.0001) (Reich et al, 2019). Characterization of psoriatic skin lesions demonstrated differential pharmacodynamic (PD) effects between guselkumab and secukinumab on the ratio of tissue-resident memory cells to regulatory T cells and identified specific CD64^+^ mononuclear phagocytes as the major IL-23–expressing subset of cells in psoriatic skin lesions (Mehta et al, 2021). The objective of this analysis is to extend these findings and evaluate the PD effects of guselkumab versus secukinumab on psoriatic skin gene expression and psoriasis-associated serum proteins and to evaluate their relationship with long-term clinical efficacy. To our knowledge, analyses comparing the long-term PD and biological effects of blocking IL-23 with those of IL-17A in patients with psoriasis have not been previously reported.

## Results

### Study population and demographics

ECLIPSE was a phase 3, randomized, double-blind, multicenter, placebo- and active-comparator–controlled study of guselkumab in patients with moderate-to-severe psoriasis. Of the 1048 eligible patients enrolled, 534 were assigned to receive guselkumab, and 514 were assigned to receive secukinumab (Reich et al, 2019). Baseline demographic and disease characteristics for a subset of patients (156 in the guselkumab-treated group and 146 in the secukinumab-treated group) were further analyzed (cytokine PD, gene expression) and were generally balanced across treatment groups and representative of the overall ECLIPSE study population (Table 1).

**Table 1:** Baseline Demographic and Disease Characteristics of Patients in the Olink Proteomics Analysis

Characteristic	Guselkumab-Treated Group n = 156	Secukinumab-Treated Group n = 146	Overall ECLIPSE study population n = 1048
Age, mean (SD), y	47.3 (13.5)	46.1 (13.4)	45.8 (13.6)
Male, n (%)	111 (71.1)	98 (67.1)	707 (67.5)
Race, n (%)			
White	147 (94.2)	139 (95.2)	979 (93.4)
Black or African American	1 (0.6)	3 (2.0)	16 (1.5)
Asian	8 (5.1)	0	30 (2.9)
Other	0	4 (2.7)	23 (2.2)
BMI, mean (SD), kg/m^2^	30.5 (7.3)	30.3 (6.5)	29.9 (6.7)
Disease duration, mean (SD), y	19.6 (12.1)	18.4 (12.5)	18.4 (12.4)
Baseline body surface area involvement %, mean (SD)	24.9 (14.2)	23.4 (24.3)	24.1 (13.7)
Baseline PASI, mean (SD)	20.7 (8.2)	19.5 (7.2)	20.0 (7.5)

Abbreviation: BMI, body mass index.

### Outcomes

#### PD effects on circulating cytokines

**Figure 1:**
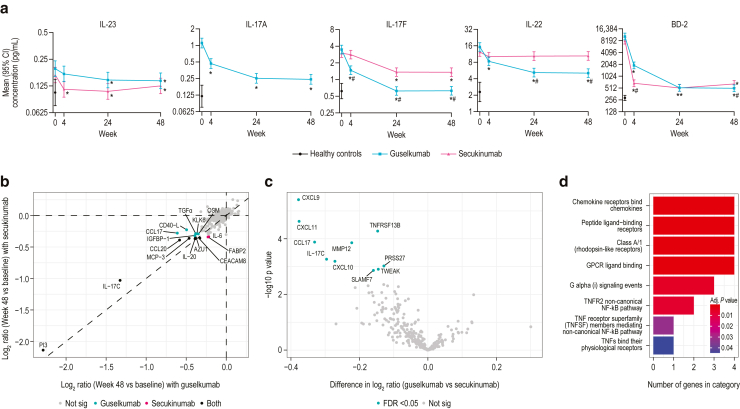
**Assessment of serum analyte levels in patients treated with guselkumab and secukinumab.** (**a**) Mean concentration (pg/ml) of IL-23, IL-17A, IL-17F, IL-22, and BD-2 serum levels in guselkumab (n = 100) and secukinumab (n = 100) treatment groups. Twenty-five healthy controls were included in this analysis. Error bars represent 95% CIs. Analyte levels from healthy control sera are presented in the lower left corner for each protein. Asterisks (∗) denote a statistically significant difference from baseline; pound signs (#) denote a statistically significant difference between guselkumab and secukinumab treatment (all *P* < .05). IL-17A levels are not displayed for secukinumab because antibody-bound IL-17A interferes with assessment of free IL-17A levels. (**b**) Log_2_ ratio (week 48 vs week 0) of guselkumab (n = 156) and secukinumab (n = 146) on serum proteins as measured by Olink assay. Analytes with significant pharmacodynamic effect with both treatments (black), with guselkumab (green), and with secukinumab (red) are displayed. (**c**) Comparison of analyte levels with guselkumab versus secukinumab treatment. Analytes with significant differences for guselkumab are noted in green; nonsignificant differences are noted in gray. (**d**) Reactome pathway analysis of 11 serum analytes better normalized in the guselkumab treatment group. BD-2, β-defensin-2; CI, confidence interval; FDR, false discovery rate; GPCR, G protein–coupled receptor.

The effect of treatment with guselkumab and secukinumab over time on serum levels of the cytokines IL-23, IL-17A, IL-17F, IL-22, and β-defensin-2 (BD-2) was evaluated (Figure 1a and Table 2, Table 3, Table 4, Table 5, Table 6). IL-23 was reduced significantly with guselkumab treatment at weeks 24 and 48 versus at baseline (week 0) and with secukinumab treatment at weeks 4, 24, and 48 versus at baseline (all *P* < .05). In line with published results, secukinumab more rapidly reduced IL-23 at 4 weeks, compared with guselkumab (Mehta et al, 2021). At weeks 4, 24, and 48, guselkumab treatment led to significantly reduced serum IL-17A levels compared with the levels at baseline. However, free IL-17A could not be distinguished from secukinumab-bound IL-17A in the assay; therefore, data for reduction of serum IL-17A levels with secukinumab treatment are not shown.

**Table 2:** Serum IL-23A Comparisons in Patients Treated with Guselkumab or Secukinumab

Treatment	Week	Avg Treatment Group IL-23A Log2conc (pg/ml)	Avg HC IL-23A Log2conc (pg/ml)	*P*-Value	Log2ratio vs HC
GUS	0	−2.370397548	−3.246324656	.003851^1^	0.875927108
GUS	4	−2.579127111	−3.246324656	.018075^1^	0.667197545
GUS	24	−2.758378704	−3.246324656	.086941	0.487945952
GUS	48	−2.818471928	−3.246324656	.135925	0.427852728
SEC	0	−2.572347183	−3.246324656	.01934^1^	0.673977473
SEC	4	−3.122076846	−3.246324656	.664485	0.12424781
SEC	24	−3.240175902	−3.246324656	.982629	0.006148754
SEC	48	−2.980382305	−3.246324656	.364252	0.26594235

Abbreviations: Avg, average; conc, concentration; Grp, group; GUS, guselkumab; HC, healthy control; SEC, secukinumab; vs, versus.

Statistics results from the comparisons are shown.

1 Welch *t*-test *P*-values <0.05.

**Table 3:** Serum IL-17A Concentrations in Patients Treated with Guselkumab

Treatment	Week	Avg Treatment Group IL-17A Log2conc (pg/ml)	Avg HC IL-17A Log2conc (pg/ml)	*P*-Value	Log2ratio vs HC
GUS	0	0.145772854	−3.062695292	9.78E-11	3.208468146
GUS	4	−1.095844909	−3.062695292	4.03E-06	1.966850383
GUS	24	−1.986971817	−3.062695292	.005180201	1.075723475
GUS	48	−2.061706827	−3.062695292	.008698572	1.000988464

Abbreviations: Avg, average; conc, concentration; Grp, group; GUS, guselkumab; HC, healthy control; vs, versus.

IL-17A levels are not displayed for secukinumab because antibody-bound IL-17A interferes with the assessment of free IL-17A levels.

**Table 4:** Serum IL-17F Comparisons in Patients Treated with Guselkumab or Secukinumab

Treatment	Week	Avg Treatment Group IL-17F Log2conc (pg/ml)	Avg HC IL-17F Log2conc (pg/ml)	*P*-Value	Log2ratio vs HC
GUS	0	1.799332011	−0.691883724	3.31E-12	2.491215735
GUS	4	0.563581197	−0.691883724	2.39E-05	1.255464922
GUS	24	−0.683238429	−0.691883724	.973063085	0.008645295
GUS	48	−0.670057224	−0.691883724	.932420113	0.0218265
SEC	0	1.582500142	−0.691883724	4.75E-11	2.274383866
SEC	4	1.494891141	−0.691883724	1.55E-10	2.186774866
SEC	24	0.457503525	−0.691883724	5.00E-05	1.149387249
SEC	48	0.433354001	−0.691883724	6.43E-05	1.125237725

Abbreviations: Avg, average; conc, concentration; Grp, group; GUS, guselkumab; HC, healthy control; SEC, secukinumab; vs, versus.

**Table 5:** Serum IL-22 Comparisons in Patients Treated with Guselkumab or Secukinumab

Treatment	Week	Avg Treatment Group IL-22 Log2conc (pg/ml)	Avg HC IL-22 Log2conc (pg/ml)	*P*-Value	Log2ratio vs HC
GUS	0	3.931931452	1.198637352	2.67E-10	2.7332941
GUS	4	3.029944805	1.198637352	1.30E-06	1.831307453
GUS	24	2.403793282	1.198637352	.00049697	1.20515593
GUS	48	2.35715838	1.198637352	.000741152	1.158521028
SEC	0	3.623752611	1.198637352	4.63E-09	2.425115259
SEC	4	3.348415998	1.198637352	6.19E-08	2.149778646
SEC	24	3.357549244	1.198637352	5.70E-08	2.158911892
SEC	48	3.386892767	1.198637352	4.34E-08	2.188255415

Abbreviations: Avg, average; conc, concentration; Grp, group; GUS, guselkumab; HC, healthy control; SEC, secukinumab; vs, versus.

**Table 6:** Serum BD-2 Comparisons in Patients Treated with Guselkumab or Secukinumab

Treatment	Week	Avg Treatment Group BD-2 Log2conc (pg/ml)	Avg HC BD-2 Log2Conc (pg/ml)	*P*-Value	Log2ratio vs HC
GUS	0	13.34864101	8.167054696	4.90E-14	5.181586318
GUS	4	10.9111621	8.167054696	1.60E-13	2.744107402
GUS	24	9.023421849	8.167054696	.236041845	0.856367153
GUS	48	8.983609601	8.167054696	.304972027	0.816554906
SEC	0	12.99886891	8.167054696	4.90E-14	4.839354007
SEC	4	9.403530987	8.167054696	.006414729	1.236476291
SEC	24	9.007794381	8.167054696	.27052694	0.836521839
SEC	48	9.344418366	8.167054696	.012715225	1.177363671

Abbreviations: Avg, average; BD-2, β-defensin-2; conc, concentration; Grp, group; GUS, guselkumab; HC, healthy control; SEC, secukinumab; vs, versus.

Reductions in other serum cytokine levels were also compared for treatment with guselkumab versus secukinumab. IL-17F serum levels showed significantly greater reduction with guselkumab than with secukinumab at weeks 4, 24, and 48 (all *P* < .05) and reached levels near those of healthy controls with guselkumab at weeks 24 and 48. Similarly, IL-22 serum levels showed significantly greater reduction with guselkumab than with secukinumab at weeks 24 and 48 (both *P* < .05). These findings demonstrate that treatment with guselkumab was associated with more rapid and sustained reductions in serum IL-17F and IL-22 than treatment with secukinumab. However, significantly greater reduction in BD-2 levels was observed with secukinumab than with guselkumab at week 4 (*P* < .05), but at week 48, the degree of reduction was significantly greater with guselkumab than with secukinumab (*P* < .05).

**Figure 2:**
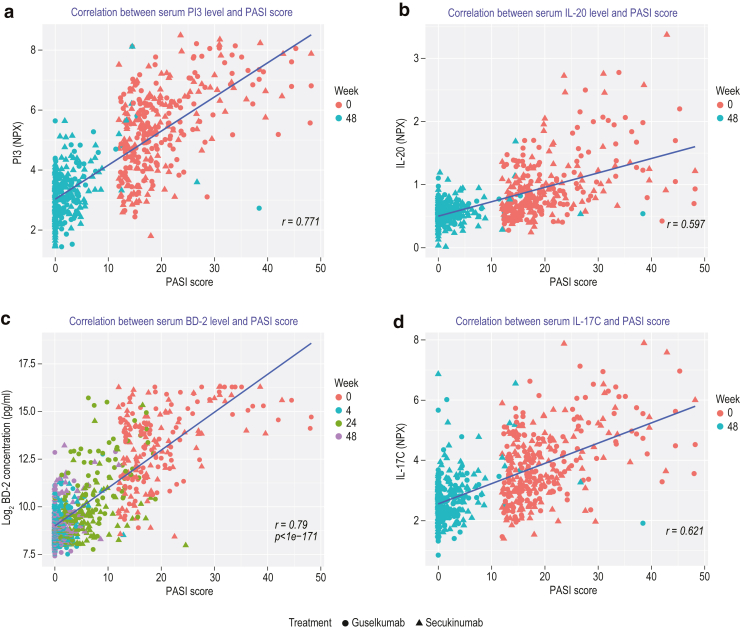
**Correlation of serum analyte levels with PASI scores.** Serum levels of (**a**) PI3 and (**b**) IL-20, assessed by Olink; (**c**) BD-2, measured by an internally developed assay; and (**d**) IL-17C, assessed by Olink with PASI scores for guselkumab (green) and secukinumab (red). BD-2, β-defensin-2; NPX, Normalized Protein eXpression.

Sera from patients collected at baseline and week 48 were also analyzed using the Olink proteomic platform (276 analytes). Overall, PD effects on the serum proteomic profile at week 48 were similar between guselkumab and secukinumab (r = 0.911, *P* < 1 × 10^−6^). Similarly, overall PD effects were also observed between patients who achieved versus those who did not achieve absolute PASI <3 response from weeks 20 to 48 with guselkumab (r = 0.893, *P* < 1 × 10^−6^) and secukinumab (r = 0.898, *P* < 1 × 10^−6^). Only serum PI3 was differentially modulated between responders (5.0-fold reduction) and inadequate responders (3.2-fold reduction) after treatment with secukinumab but not with guselkumab (false discovery rate [FDR] < 0.05). At week 48, guselkumab treatment led to significant reductions of 14 proteins (fold change > 1.25 and FDR < 0.05) from baseline, whereas 9 proteins were significantly reduced with secukinumab (Figure 1b). Among these, AZU1, CCL20, CEACAM8, IL-17C, IL-20, MCP-3, and PI3 were reduced in both groups; CCL17, CD40-L, IGFBP-1, KLK6, oncostatin M, and TGFα were reduced only in the guselkumab-treated group, and FABP2 and IL-6 were reduced only in the secukinumab-treated group (Table 7). CCL20, IL-6, IL-20, IL-17C, and PI3 were previously found to be elevated in patients with psoriasis (Navrazhina et al, 2022). In addition, PI3, IL-20, BD-2, and IL-17 levels correlated with PASI scores (Figure 2). Direct comparison of analyte levels showed significantly (FDR <0.05) greater reductions in CXCL9, CXCL10, CXCL11, CCL17, IL-17C, matrix metalloproteinase 12, PRSS27, SLAMF7, TNFRSF13B, and TWEAK at week 48 with guselkumab treatment than with secukinumab treatment (Figure 1c). Notably, these proteins are enriched for Gene Ontology pathways involved in chemokine, anti-TNF, and immune-relevant signaling pathways (Figure 1d).

**Table 7:** Olink Analysis Comparing Baseline with Week 48

Assay	Guselkumab Log2 Ratio	Secukinumab Log2 Ratio	Guselkumab Versus Secukinumab Log2 Ratio	Guselkumab FDR	Secukinumab FDR	Guselkumab Versus Secukinumab FDR
PI3	−2.29	−2.14	−0.15	2.20E-113	4.08E-102	0.390155
IL-17C	−1.33	−1.03	−0.3	7.62E-65	1.12E-43	0.021414
CCL17	−0.61	−0.28	−0.33	4.02E-20	0.000135	0.006414
CCL20	−0.58	−0.39	−0.19	2.62E-10	0.000135	0.414802
CD40-L	−0.5	−0.23	−0.27	0.00022	0.202236	0.416305
MCP-3	−0.46	−0.36	−0.1	8.91E-16	3.68E-09	0.519661
IL-20	−0.39	−0.37	−0.02	9.34E-81	1.63E-72	0.656919
AZU1	−0.39	−0.35	−0.03	0.000101	0.001228	0.939023
IGFBP-1	−0.39	−0.32	−0.07	0.000101	0.004006	0.864045
TGF-α	−0.38	−0.29	−0.1	8.16E-10	2.71E-05	0.578571
KLK6	−0.37	−0.29	−0.09	2.72E-32	1.07E-19	0.221203
OSM	−0.35	−0.29	−0.06	3.01E-06	0.000379	0.852972
CEACAM8	−0.33	−0.36	0.03	3.31E-06	2.50E-06	0.931987
FABP2	−0.23	−0.34	0.11	0.002869	2.73E-05	0.578571
IL-6	−0.22	−0.34	0.12	0.001222	2.77E-06	0.519661

Abbreviations: FDR, false discovery rate; OSM, oncostatin M.

#### Skin biopsy gene expression analysis

**Figure 3:**
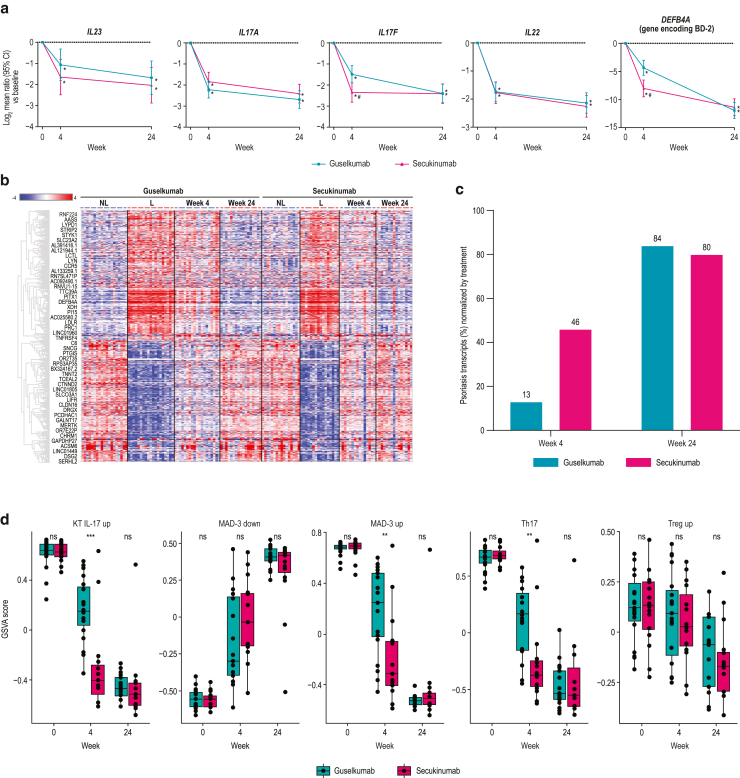
**Transcriptional changes in psoriatic skin during treatment with guselkumab and secukinumab.** Lesional and nonlesional skin biopsies were collected from 19 patients treated with guselkumab and 16 patients treated with secukinumab at baseline. Lesional skin was collected at week 4 from 19 guselkumab-treated patients and 15 secukinumab-treated patients and at week 24 from 17 guselkumab-treated patients and 15 secukinumab-treated patients. (**a**) Changes in transcript levels of *IL23*, *IL17A*, *IL17F*, *IL22*, and *DEFB4A* expressed as mean log_2_ ratio versus baseline (week 0) by RNAseq. Error bars represent 95% CIs. Asterisks denote a statistically significant difference from baseline; pound signs denote a statistically significant difference between guselkumab and secukinumab treatment (all *P* < .05). (**b**) Heatmap of 3575 differentially regulated psoriasis transcripts from lesional and nonlesional tissue at baseline and after guselkumab and secukinumab treatment in lesional tissue at Weeks 4 and 24. (**c**) Percentage of psoriasis-associated transcripts normalized at Weeks 4 and 24 after guselkumab and secukinumab treatment. (**d**) Box and whisker plots (generated using R boxplot, where the lower and upper hinges correspond to the first and third quartiles, respectively) illustrating mean GSVA in guselkumab and secukinumab expression profiles at baseline, Week 4, and Week 24. Gene sets used for analysis were curated gene lists from KTs after IL-17A treatment (KT IL-17 upregulated), MAD-3 upregulated and downregulated signatures, and Th17 and Treg cell signatures. Asterisks denote *P*-values—∗∗∗*P* < .001, ∗∗*P* < .01, and ∗*P* < .05; ns = *P* > .05—as determined by Kruskal–Wallis testing. IL-17 up denotes IL-17 upregulated, MAD-1 down denotes MAD-3 downregulated, MAD-3 up denotes MAD-3 upregulated, and Treg up denotes Treg upregulated. BD-2, β-defensin-2; CI, confidence interval; GSVA, gene set variation analysis; KT, keratinocyte; L, lesional; MAD-3, meta-analysis–derived 3; NL, nonlesional; ns, nonsignificant; RNAseq, RNA sequencing; Th17, T helper 17; Treg, regulatory T cell.

**Figure 4:**
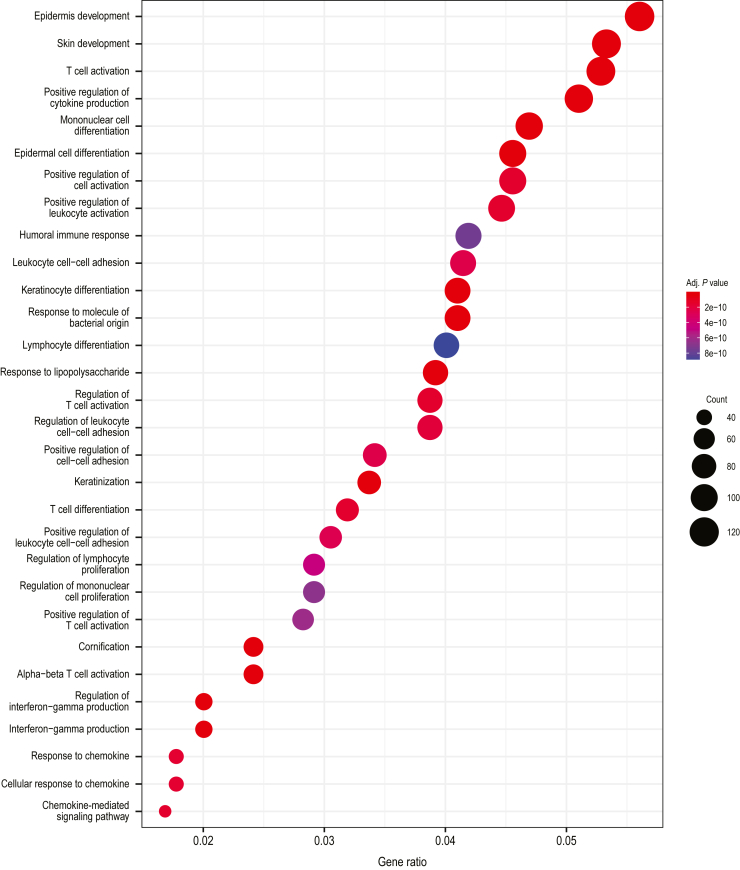
**Skin transcriptomic pathways associated with psoriatic skin.** Gene Ontology biological process term enrichment analysis for the 3575 differentially expressed psoriasis transcripts from baseline comparison between lesional and nonlesional psoriasis samples from all treatment groups, that is, 35 week 0 lesional samples versus 35 nonlesional paired patient samples. Adj. *P*-value denotes adjusted *P*-value. Size of dot represents the number of transcripts in that category; color is adjusted for *P*-value.

Transcriptomic analyses of biopsies from lesional and nonlesional skin at baseline and weeks 4 and 24 of treatment were performed using RNA sequencing (RNAseq). Lesional and nonlesional skin biopsies were collected from 19 guselkumab-treated patients and 16 secukinumab-treated patients at baseline. Lesional skin was collected at week 4 from 19 guselkumab-treated patients and 15 secukinumab-treated patients and at week 24 from 17 and 15 patients, respectively. Both guselkumab and secukinumab treatment significantly reduced the expressions of *IL23*, *IL17A*, *IL17F*, *IL22*, and *DEFB4A* (gene encoding BD-2) at weeks 4 and 24 compared with those at baseline (all *P* < .05) (Figure 3a). Levels of reduction in *IL17F* and *DEFB4A* expression from baseline were significantly greater with secukinumab than with guselkumab at week 4 (both *P* < .05) but were similar at week 24. To define a psoriasis transcriptome, gene expression profiles for baseline lesional and nonlesional skin biopsy samples from 35 patients (guselkumab, n = 19; secukinumab, n = 16) were compared. A total of 3575 differentially expressed transcripts (fold change > 2, FDR < 0.05) were identified as psoriasis-associated transcripts. Across both treatment groups at baseline, transcripts expressed in lesional versus nonlesional skin were enriched in pathways involved in epidermal/keratinocyte differentiation, cytokine/chemokine signaling, inflammation, and regulation of immune response (T-cell and leukocyte activation/migration) (Figure 4), consistent with previous studies (Tian et al, 2012).

Changes in expression of psoriasis-associated transcripts after treatment with guselkumab and secukinumab were assessed only for differentially expression genes, as illustrated in Figure 3b, and quantified, as shown in Figure 3c. At week 4, the proportion of psoriasis-associated transcripts showing normalization of expression with >75% improvement from baseline was significantly higher in the secukinumab-treated group than in the guselkumab-treated group (46 vs 13%, respectively) (Figure 3c). However, by week 24, the proportions of psoriasis transcriptome genes that showed normalized levels of expression were similar for the secukinumab- and guselkumab-treated groups (80 vs 84%, respectively).

To further explore the normalization of relevant psoriasis gene sets with treatment, we employed gene set variation analysis using the meta-analysis derived-3 psoriasis transcriptome gene sets and curated genes sets for Th17 cells, IL-17-stimulated keratinocytes, and regulatory T cell responses (Krueger et al, 2019; Tian et al, 2012). In concordance with results shown earlier, secukinumab showed significantly greater normalization of keratinocyte IL-17 upregulated, meta-analysis derived-3 upregulated, and Th17 gene sets than guselkumab at week 4 (Figure 3d). However, at week 24, guselkumab and secukinumab showed similar levels of normalization of these gene sets.

**Figure 5:**
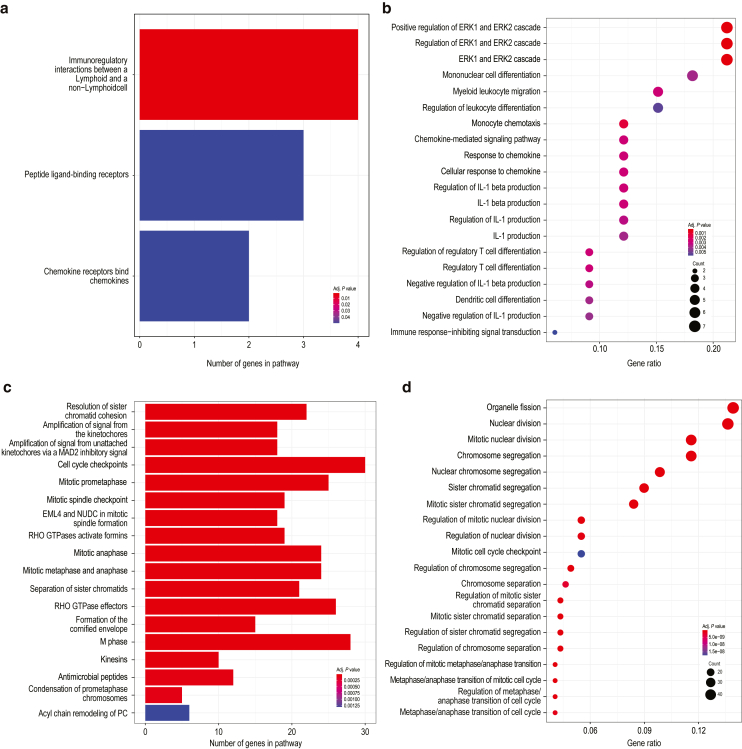
**Skin transcriptomic pathway differences between the guselkumab and secukinumab treatment groups at week 4.** (**a**) Reactome pathway analysis and (**b**) Gene Ontology biological process term enrichment analysis for 62 transcripts better normalized by guselkumab than by secukinumab at week 4. (**c**) Reactome pathway analysis and (**d**) Gene Ontology biological process term enrichment analysis for 530 transcripts better normalized by secukinumab than by guselkumab at week 4. Size of dot represents the number of transcripts in that category; color is adjusted for *P*-value.

**Figure 6:**
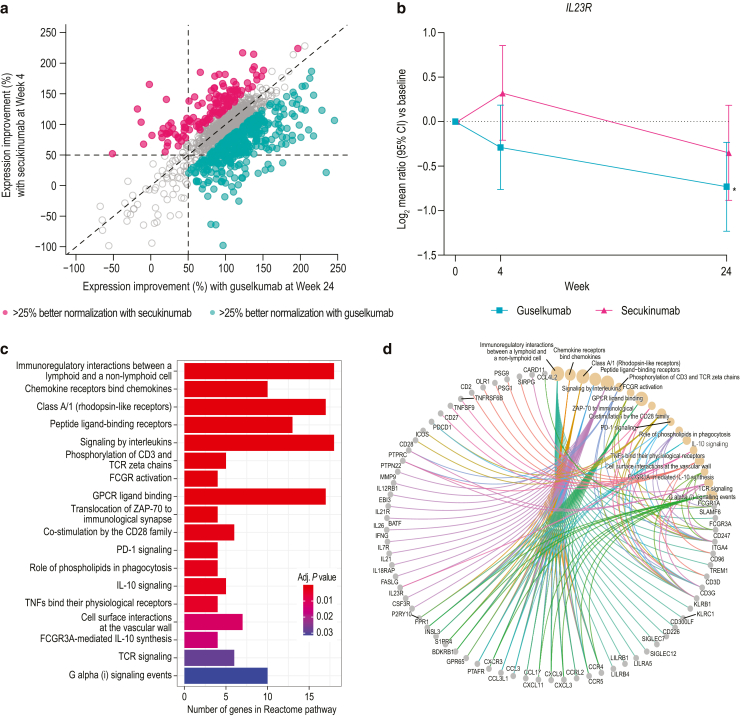
**Differential transcriptional changes in psoriatic skin during treatment with guselkumab and secukinumab at week 24.** (**a**) Percentage improvement of expressed psoriasis gene transcripts for guselkumab and secukinumab. Transcripts that were better normalized (>50% improvement and >25% difference between treatment) in the guselkumab (green) and secukinumab (red) treatment groups are highlighted. (**b**) Changes in transcript levels of *IL23R* expressed as mean log_2_ ratio versus baseline (week 0) by RNAseq. Error bars represent 95% CIs. Asterisk denotes statistically significant difference from baseline (*P* < .05). (**c**) Reactome pathway analysis of the 383 transcripts better normalized in the guselkumab treatment arm. (**d**) Cnetplot depicts the connection of individual genes from 383 transcripts to individual enriched pathways in panel **c** as a network. CI, confidence interval; FCGR, Fc gamma receptor; GPCR, G protein–coupled receptor; RNAseq, RNA sequencing.

**Figure 7:**
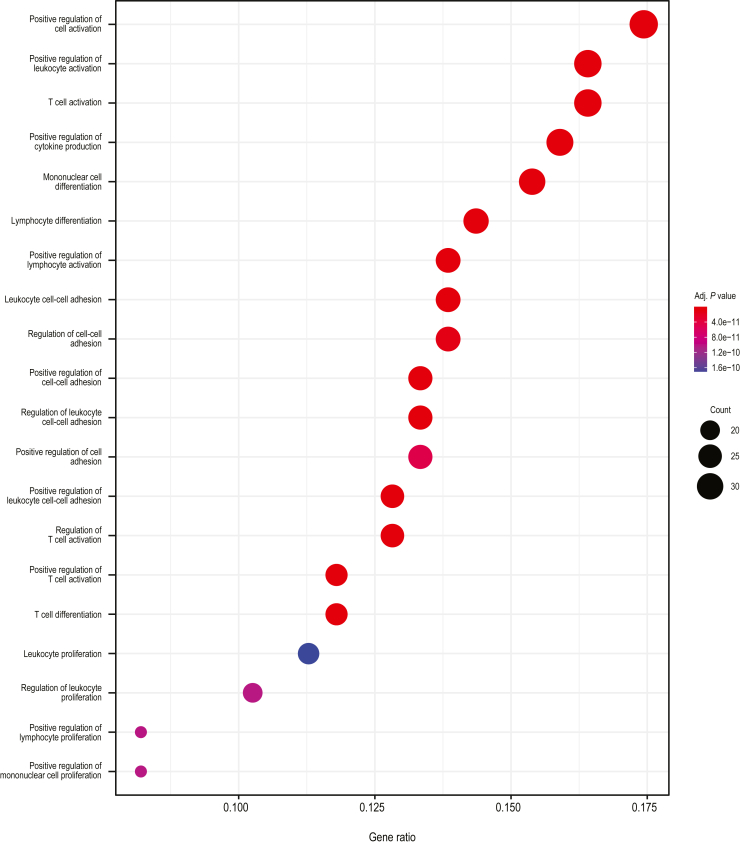
**Skin transcriptomic pathway differences between the guselkumab and secukinumab treatment groups at week 24.** Gene Ontology biological process term enrichment analysis for 383 transcripts better regulated by guselkumab than by secukinumab at week 24. Size of dot represents number of transcripts in that category; color is adjusted for *P*-value. Adj. P-value denotes adjusted *P*-value.

Differences were observed with respect to the numbers and types of psoriasis-associated transcripts that were normalized by treatment. At week 4, more transcripts were normalized (>50% improvement and >25% difference between treatments) with secukinumab than with guselkumab (530 vs 62 transcripts, respectively). Pathway analysis of transcripts showing greater normalization with secukinumab indicated enrichment of processes related to mitosis and cell proliferation, whereas transcripts showing greater normalization with guselkumab demonstrated enrichment of immune-related processes (Figure 5). In contrast, more transcripts were normalized with guselkumab than with secukinumab at week 24 (383 vs 124 transcripts, respectively) (Figure 6a). Expression of *IL23R* was significantly reduced at week 24 compared with that at baseline in the guselkumab-treated group (*P* < .05) but not in the secukinumab-treated group (Figure 6b). However, numerically greater but not statistically significant reduction in *IL23R* expression was observed with guselkumab than with secukinumab treatment, perhaps due to the limited number of biopsy samples and/or sample variabilities. Consistent with data at week 4, pathway analysis of the 383 transcripts that showed greater normalization with guselkumab at week 24 revealed enrichment of immune-related processes (Figures 6c and 7), including immune signaling and regulation of T-cell signaling. Normalized genes mapping to these pathways are displayed in Figure 6d. Analysis of the 124 transcripts better normalized by secukinumab showed enrichment only of genes associated with the Reactome pathway post-translational modification: synthesis of glycosylphosphatidylinositol-anchored proteins (data not shown).

## Discussion

The ECLIPSE trial was the first head-to-head, phase 3, randomized study to compare the safety and efficacy of an IL-23p19-subunit inhibitor (guselkumab) with those of an IL-17A inhibitor (secukinumab) in patients with moderate-to-severe plaque-type psoriasis (Reich et al, 2019). The proportions of patients achieving PASI 90 response between weeks 3 and 12 were higher in the secukinumab-treated group than in the guselkumab-treated group; between weeks 16 and 20, similar proportions of responders were observed in both groups. However, after week 20, greater proportions of patients achieved PASI 90 response with guselkumab treatment, with the difference at week 48 (primary study endpoint) achieving statistical significance (84 vs 70%, *P* < .0001) (Reich et al, 2019; Yiu et al., 2022). In turn, this analysis compares the PD and biologic effects of blocking IL-23 with those of IL-17A in patients with psoriasis. The results provide a mechanistic basis for the differences in efficacy observed between guselkumab and secukinumab through approximately 1 year of treatment.

Consistent with IL-17A acting directly on keratinocytes to induce epidermal acanthosis in psoriasis as an effector cytokine (Ha et al, 2014), secukinumab was associated with faster and greater normalization of expression of transcripts dysregulated in psoriasis skin lesions at week 4. Pathway analyses demonstrated that inhibition of IL-17A normalized the genes associated with cell proliferation and mitosis pathways. These findings may explain the generally faster clinical response observed in secukinumab-treated patients in the early weeks of treatment. However, the induction dosing regimen (weekly secukinumab injections through week 4) may, in part, also contribute to the faster clinical and PD responses observed with secukinumab at earlier time points.

Although guselkumab showed fewer differentially modulated transcripts at week 4, transcripts uniquely normalized by guselkumab were related to immune processes. Pathways associated with T cells and other immune cell subsets also demonstrated greater normalization at week 24 with guselkumab. In particular, expression of *IL23R*, which encodes the IL-23 receptor, was significantly reduced at week 24 with guselkumab but not with secukinumab. This finding suggests that IL-23p19 inhibition but not IL-17A inhibition reduces the numbers of IL-23-responsive cells (eg, Th17 and Tc17 cells). Furthermore, reduced expression of *IL23R* is also consistent with the concept that IL-23 signaling regulates IL-23 receptor expression (Teng et al, 2015). IL-23 receptor signaling is also required for terminal differentiation of Th17 cells (Boniface et al, 2008; McGeachy et al, 2009) and cutaneous tissue-resident memory cell 17 survival (Whitley et al, 2022). In turn, this finding of reduced *IL23R* expression provides further insight into the sustained long-term impact of guselkumab treatment on disease modulation.

IL-17A, IL-17F, and IL-22 function downstream of IL-23, and reductions in their serum levels and gene expressions in psoriatic skin lesions were observed in response to guselkumab treatment. These findings further support IL-23 as a dominant regulatory cytokine that modulates the expression of these cytokines and expansion and maintenance of CD4^+^ Th17, T helper 22, IL-10 nonpathogenic Th17 cells (Kim et al, 2021), and other CD8^+^ T-cell subsets that produce them (Gordon et al, 2019; Sofen et al, 2014; Teng et al, 2015). Reductions in IL-17A/F and IL-22, in turn, lead to downstream reductions in BD-2 because keratinocyte expression of BD-2 is directly induced by these cytokines (Liang et al, 2006). Whereas inhibition of IL-17A, an effector cytokine, was associated with more rapid clinical improvement, inhibition of IL-23 demonstrated greater and more sustained reductions of IL-23/Th17 pathway cytokines, including IL-17F and IL-22. In particular, greater reductions in serum IL-17F with guselkumab treatment may also have clinical relevance, as supported by recent phase 3 data demonstrating that the dual IL-17A/IL-17F inhibitor, bimekizumab, provided greater skin clearance than did secukinumab (IL-17A inhibitor) (Reich et al, 2021b), although other factors may also contribute to this difference.

The rapidity of reduction in serum IL-17F and IL-22 levels was generally comparable between the guselkumab and secukinumab cohorts. However, guselkumab exhibited a greater magnitude of change in serum IL-17F and IL-22 levels at given time points. One possible explanation for this finding may be related to differences in the ability of guselkumab and secukinumab to reduce the numbers of cells that serve as the source of IL-17F and IL-22 (eg, the differential impact between guselkumab and secukinumab on tissue-resident memory cells [Mehta et al, 2021]). However, the exact mechanism for the rapid reduction in serum IL-17F and IL-22 levels with guselkumab treatment is unknown.

A number of serum analytes identified by Olink analysis, including PI3, IL-17C, CCL20, IL-20, IL-6, CXCL9, and CXCL10, have been previously identified as being elevated in serum samples of patients with psoriasis (Navrazhina et al, 2022). Although PI3, BD-2, and IL-20 showed the highest correlation with PASI in our dataset, these mediators do not seem to play a central role in psoriasis. These antimicrobial proteins and cytokine might be a sign of IL-17 signaling. There are studies to show that IL-17 promotes IL-20 expression in keratinocytes and synovial fibroblasts. An anti–IL-20 antibody evaluated in a phase 1 trial of in patients with psoriasis did not further progress into clinical development (NCT01261767) (Blumberg et al, 2001). The current analysis found that serum levels of PI3, IL-17C, and IL-20 were reduced at week 48 compared with the levels at baseline after treatment with either guselkumab or secukinumab. However, serum levels of IL-17C, CXCL9, and CXCL10 showed significantly greater reductions with guselkumab than with secukinumab treatment. Furthermore, Reactome pathway proteomic analyses of patient sera and gene expression profiling of psoriatic skin lesions generally yielded similar results. Overall, these data are consistent with the concept of systemic inflammation in psoriasis and the potential for guselkumab having greater long-term impact than secukinumab on normalizing inflammation in psoriasis.

Limitations to these analyses should be acknowledged. The sample size of the biomarker substudy was small, and the number of skin biopsies available for analysis was limited. In addition, clinical differences in efficacy between guselkumab and secukinumab and the kinetics of molecular responses could be influenced by differences in dosing regimens and time to reach steady state levels in the blood (Bruin et al, 2017; Yao et al, 2018).

In summary, this analysis utilized molecular approaches to improve the understanding of the mechanistic basis for the differences in the clinical efficacy profiles observed after treatment of psoriasis with guselkumab and secukinumab. Greater reductions in serum cytokine levels (IL-17F and IL-22) were seen with guselkumab treatment at week 4 and were sustained through the week 48 primary endpoint, at which guselkumab demonstrated superiority in achieving a PASI 90 response (Reich et al, 2019). In contrast, at week 4, more rapid normalization of the psoriasis transcriptome in lesional skin was observed with secukinumab than with guselkumab, consistent with clinical findings; however, by week 24, the degree of transcriptional normalization was essentially equivalent. Results from this analysis at both early and later time points suggest that guselkumab has greater impact on modulating genes associated with immune-related processes in psoriasis than secukinumab, consistent with prior findings that guselkumab better normalizes the ratio of regulatory T cell:tissue-resident memory in psoriatic skin. Taken together, these findings provide additional insights into the differential mechanisms and impact of IL-23 versus IL-17A blockade in psoriasis.

## Materials and Methods

### Patients and study design

**Figure 8:**
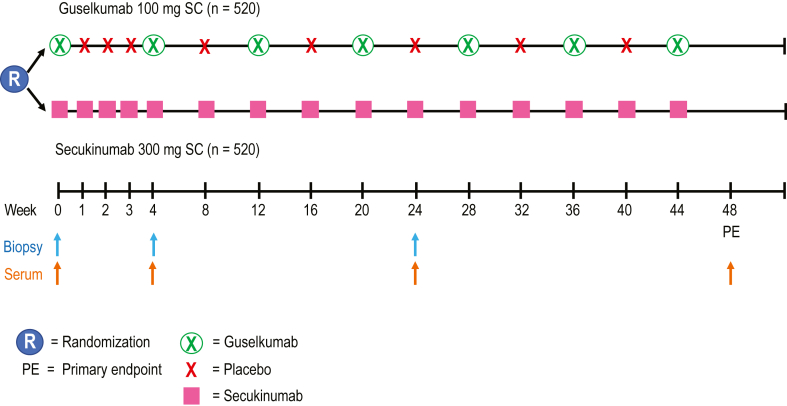
**ECLIPSE study design.** SC, subcutaneous.

**Figure 9:**
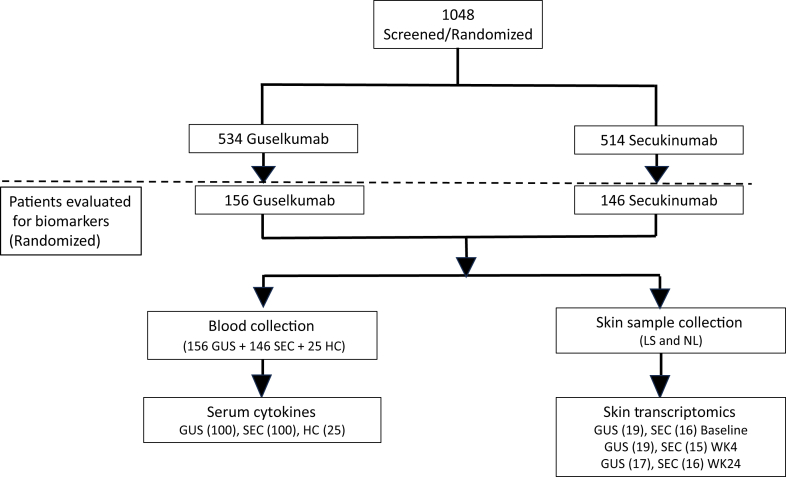
**Participant flow diagram.** GUS, guselkumab; HC, healthy control; LS, lesional; NL, nonlesional; SEC, secukinumab; WK, week.

ECLIPSE (ClinicalTrials.gov identifier: NCT03090100) was a phase 3, randomized, double-blind, multicenter, placebo- and comparator-controlled trial conducted at 142 outpatient centers. Study design for the ECLIPSE trial was previously published (Figure 8) (Reich et al, 2019). In brief, adults aged 18 years or older with moderate-to-severe plaque psoriasis (PASI ≥12, Investigator’s Global Assessment score ≥3, and body surface area involvement ≥10% for ≥6 months) and who were candidates for phototherapy or systemic therapy were eligible (Table 1 and Figure 9). Those who had received any therapeutic agent directly targeting IL-12/23p40, IL-17A, IL-17R, or IL-23p19 within the prior 6 months or any systemic immunosuppressant within 4 weeks prior to first study drug administration were ineligible.

Patients were randomized (1:1) to receive guselkumab 100 mg by subcutaneous injection at weeks 0, 4, and 12 and then every 8 weeks until week 44 or secukinumab 300 mg (as two 150-mg subcutaneous injections) at weeks 0, 1, 2, 3, and 4 and every 4 weeks thereafter until week 44 (Reich et al, 2019). The study protocol was approved by an institutional review board or ethics commitee at each site (Supplementary Table S1). Sterling Institutional Review Board was a central United States institutional review board for the ECLIPSE sites. Patients provided written informed consent before study initiation.

### Biomarker assessments

#### Blood sample collection and analysis

Serum was isolated from blood samples collected at weeks 0, 4, 24, and 48 for evaluation of potential biomarkers of PD response to treatment. Healthy control sera were procured separately (BioIVT) (Table 8). Serum IL-17A, IL-17F, IL-22, and IL-23 levels were analyzed using Singulex Errena high-sensitivity immunoassays (EMD Millipore) (Sofen et al, 2014); BD-2 levels were analyzed using a custom Mesoscale Discovery (Meso Scale Diagnostics LLC) immunoassay, following manufacturer’s instructions. Olink multiplex immunoassay analysis (Olink Proteomics) was conducted by the Olink laboratory on a subset of sera (guselkumab, n = 156; secukinumab, n = 146) representative of the ECLIPSE patient population using the Olink inflammation (92 analytes) and cardiovascular (184 analytes) panels.

**Table 8:** Demographic Characteristics of Healthy Controls

Characteristic	Healthy Controls n = 25
Age, mean (SD), y	42.2 (16.9)
Male, n (%)	13 (52)
Race, n (%)	
White	5 (20)
Black or African American	12 (48)
Asian	0 (0)
Other	8 (32)

#### Skin sample collection and analysis

Lesional and nonlesional skin biopsies were collected from 19 guselkumab-treated patients and 16 secukinumab-treated patients at baseline and 17 and 15 patients, respectively, at week 24. Biopsies were collected on a voluntary basis only from patients who provided additional consent for the optional gene expression substudy.

For gene expression analysis, one 4-mm biopsy from an active psoriasis skin lesion, representative of the patient’s overall disease severity, and one 4-mm biopsy from a nonlesional skin area >2 cm apart from the lesional site were taken at baseline. The same site of the original lesion biopsied at baseline was sampled at weeks 4 and 24. RNAseq was performed by BioProcessing Solutions (RUCDR Infinite Biologics).

### RNA isolation from skin biopsies

Bulk tissue RNA was extracted in 300 μl of Buffer RLT Plus. Tissue lysates were disrupted and homogenized using the TissueRuptor and disposable probes for 30 seconds. Each lysate was inspected and, if necessary, homogenized in additional increments of 30 seconds until uniformly homogeneous. Proteinase K and water were added, and the sample was incubated for 20 minutes at 55 °C. The homogenized RNA lysate was centrifuged at 7000 r.p.m. for 3 minutes and transferred to a new 2-ml Sarstedt tube. RNA lysates were extracted using the Qiagen RNeasy Fibrous Tissue Mini Kit, per the manufacturer’s instructions, and eluted in 50 μl of RNase-free water. RNA quality was checked with Agilent 2100 bioanalyzer before library construction.

### Library synthesis and sequencing

After extraction, RNA samples (50 ng) were reverse transcribed, and sequencing libraries were constructed using NuGEN Ovation Universal RNA-Seq System (NuGEN) according to the manufacturer’s instructions. The resulting sequencing libraries were analyzed using the Caliper LabChip GX (Caliper Life Sciences) and quantified using KAPA qPCR. Libraries were then normalized and pooled in batches of 8. Each pool of 8 samples was clustered and sequenced on an Illumina NextSeq500 instrument (Illumina) using 2 × 100 bp paired-end reads, following the manufacturer’s instructions. Each library was loaded to obtain ∼80 million reads.

### Statistical analysis

For skin biopsy RNAseq, vendor-generated raw RNAseq data were imported into OmicSoft ArrayStudio for data quality control and analysis using its RNAseq pipeline. Specifically, original FASTq files were mapped onto the Human.B38 genome version for alignment using ArrayStudio provided by Omicsoft GenCode.V29 gene model with 58,721 transcripts and 57,134 unique genes. Raw count and transcripts per million read matrices at the individual gene level were generated for downstream analysis. Differential gene expression in response to secukinumab versus guselkumab at week 4 and week 24 compared with that at baseline was evaluated by mixed effects model on log_2_-transformed ratios, with differential gene expression between lesional and nonlesional at baseline as a covariate. PD effects (log_2_ratios to baseline) were compared with 0 at each time point (week 4 and week 24) and between 2 treatment groups. Differential gene expression between paired lesional and nonlesional tissue at baseline was evaluated using voom (Law et al, 2014). On the basis of the baseline lesional versus nonlesional differential expression analysis, a psoriasis transcriptome was identified and defined as 3575 differentially expressed transcripts (1851 upregulated, 1724 downregulated) under the statistical criteria of geometric mean of fold change >2 and FDR < 0.05 among samples from all 35 patients.

To assess PD effects on the psoriasis transcriptome, the differential gene expression between lesional and nonlesional skin response to secukinumab versus guselkumab at week 4 and week 24 compared with that at baseline was evaluated using a repeated-measures analysis of covariance using linear mixed effect models in R, including treatment group and time (in weeks) as fixed factors and subject identification as random factors, plus baseline log_2_ concentration values as covariate.

Similarly, a mixed effect linear regression model with baseline gene expression and PASI response at week 48 as covariates was used to identify PD effects at different time points (vs baseline) on serum protein measurements from the Singulex assay and the Olink platform. The comparison between treatment PD effects on cytokines and serum proteins profiled using the Olink platform was analyzed using the same statistical methods as transcriptome analysis, that is, for each serum protein, a mixed effect regression model was applied on log_2_-transformed ratios to baseline, using time and treatment group as fixed effects and subject as random factors, plus baseline protein values as covariates. The relationship between serum proteins and PASI scores was assessed by Pearson correlation. Gene Ontology biological process terms were searched for enrichment. A Benjamini–Hochberg–adjusted *P*-value for false discovery <0.05 was used as a cutoff for term enrichment. The R package clusterProfiler (Yu et al, 2012) was used to perform Gene Ontology and Reactome pathway analyses.

## Ethics Statement

The study protocol was approved by an institutional review board or ethics committee at each site (list of institutional review boards and ethics committees can be found in Supplementary Table S1). Sterling Institutional Review Board was a central United States institutional review board for the ECLIPSE sites. Patients provided written informed consent prior to study initiation.

## Data Availability Statement

Raw datasets related to this article will be deposited in the National Center for Biotechnology Information’s Gene Expression Omnibus.

## ORCIDs

Andrew Blauvelt: http://orcid.org/0000-0002-2633-985X

Yanqing Chen: http://orcid.org/0000-0002-9534-6199

Patrick J. Branigan: http://orcid.org/0000-0001-9741-9067

Xuejun Liu: http://orcid.org/0000-0001-8194-3067

Samuel DePrimo: http://orcid.org/0000-0002-4195-8616

Brice E. Keyes: http://orcid.org/0009-0007-3669-3901

Monica Leung: http://orcid.org/0000-0001-6873-7015

Steven Fakharzadeh: http://orcid.org/0000-0003-0423-9388

Ya-Wen Yang: http://orcid.org/0000-0003-4249-1111

Ernesto J. Muñoz-Elías: http://orcid.org/0000-0002-2894-157X

James G. Krueger: http://orcid.org/0000-0002-3775-1778

Richard G. Langley: http://orcid.org/0000-0003-4730-2164

## Conflict of Interest

AB has served as a speaker (received honoraria) for AbbVie, Bristol Myers Squibb, Eli Lilly, Pfizer, Regeneron, and Sanofi; served as a scientific adviser (received honoraria) for AbbVie, Abcentra, Affibody, Aligos, Almirall, Alumis, Amgen, Anaptysbio, Arcutis, Arena, Aslan, Athenex, Bluefin Biomedicine, Boehringer Ingelheim, Bristol Myers Squibb, Cara Therapeutics, Dermavant, EcoR1, Eli Lilly, Escient, Evelo, Evommune, Forte, Galderma, HighlightII Pharma, Incyte, InnoventBio, Janssen, Landos, Leo, Merck, Novartis, Pfizer, Rapt, Regeneron, Sanofi Genzyme, Spherix Global Insights, Sun Pharma, TLL Pharmaceutical, TrialSpark, UCB Pharma, Vibliome, and Xencor; and has acted as a clinical study investigator (institution received clinical study funds) for AbbVie, Acelyrin, Almirall, Alumis, Amgen, Arcutis, Athenex, Boehringer Ingelheim, Bristol Myers Squibb, Concert, Dermavant, Eli Lilly, Evelo, Evommune, Galderma, Incyte, Janssen, Leo, Merck, Novartis, Pfizer, Regeneron, Sun Pharma, and UCB Pharma. YC, PJB, and ML are employees of Janssen Research & Development, LLC, and own stock or stock options in Johnson & Johnson. SF and Y-WY are employees of Janssen Pharmaceutical Companies of Johnson & Johnson, LLC, and own stock or stock options in Johnson & Johnson. XL, SD, BEK, and EJM-E are former employees of Janssen Research & Development, LLC. JGK has been a consultant or received honoraria from AbbVie, Aclaris, Allergan, Almirall, Amgen, Arena, Aristea, Asana, Aurigene, Biogen Idec, Boehringer Ingelheim, Bristol-Myers Squibb, Escalier, Galapagos, Janssen, Lilly, MoonLake Immunotherapeutics, Nimbus, Novartis, Pfizer, Sanofi, Sienna Biopharmaceuticals, Sun Pharma, Target-Derm, UCB, Valeant, and Ventyx. RGL has been a principal investigator; served on the advisory board; or has served as a speaker for AbbVie, Amgen, Boehringer Ingelheim, Celgene, Eli Lilly, Janssen, Leo Pharma, Merck, Novartis, Pfizer, Sun Pharma, and UCB Pharma.

## References

[bib1] Blauvelt A., Ferris L.K., Yamauchi P.S., Qureshi A., Leonardi C.L., Farahi K. (2017). Extension of ustekinumab maintenance dosing interval in moderate-to-severe psoriasis: results of a phase IIIb, randomized, double-blinded, active-controlled, multicentre study (PSTELLAR). Br J Dermatol.

[bib2] Blauvelt A., Papp K.A., Griffiths C.E., Randazzo B., Wasfi Y., Shen Y.K. (2017). Efficacy and safety of guselkumab, an anti-interleukin-23 monoclonal antibody, compared with adalimumab for the continuous treatment of patients with moderate to severe psoriasis: results from the phase III, double-blinded, placebo- and active comparator-controlled VOYAGE 1 trial. J Am Acad Dermatol.

[bib3] Blumberg H., Conklin D., Xu W.F., Grossmann A., Brender T., Carollo S. (2001). Interleukin 20: discovery, receptor identification, and role in epidermal function. Cell.

[bib4] Boniface K., Blom B., Liu Y.J., de Waal Malefyt R. (2008). From interleukin-23 to T-helper 17 cells: human T-helper cell differentiation revisited. Immunol Rev.

[bib5] Bruin G., Loesche C., Nyirady J., Sander O. (2017). Population pharmacokinetic modeling of secukinumab in patients with moderate to severe psoriasis. J Clin Pharmacol.

[bib6] Gordon K.B., Armstrong A.W., Foley P., Song M., Shen Y.K., Li S. (2019). Guselkumab efficacy after withdrawal is associated with suppression of serum IL-23-regulated IL-17 and IL-22 in psoriasis: VOYAGE 2 study. J Invest Dermatol.

[bib7] Ha H.L., Wang H., Pisitkun P., Kim J.C., Tassi I., Tang W. (2014). IL-17 drives psoriatic inflammation via distinct, target cell-specific mechanisms. Proc Natl Acad Sci U S A.

[bib8] Hawkes J.E., Yan B.Y., Chan T.C., Krueger J.G. (2018). Discovery of the IL-23/IL-17 signaling pathway and the treatment of psoriasis. J Immunol.

[bib9] Kim J., Lee J., Kim H.J., Kameyama N., Nazarian R., Der E. (2021). Single-cell transcriptomics applied to emigrating cells from psoriasis elucidate pathogenic versus regulatory immune cell subsets. J Allergy Clin Immunol.

[bib10] Kim J., Moreno A., Krueger J.G. (2022). The imbalance between Type 17 T-cells and regulatory immune cell subsets in psoriasis vulgaris. Front Immunol.

[bib11] Krueger J.G., Wharton K.A., Schlitt T., Suprun M., Torene R.I., Jiang X. (2019). IL-17A inhibition by secukinumab induces early clinical, histopathologic, and molecular resolution of psoriasis. J Allergy Clin Immunol.

[bib12] Langley R.G., Elewski B.E., Lebwohl M., Reich K., Griffiths C.E., Papp K. (2014). Secukinumab in plaque psoriasis--results of two phase 3 trials. N Engl J Med.

[bib13] Law C.W., Chen Y., Shi W., Smyth G.K. (2014). voom: Precision weights unlock linear model analysis tools for RNA-seq read counts. Genome Biol.

[bib14] Liang S.C., Tan X.Y., Luxenberg D.P., Karim R., Dunussi-Joannopoulos K., Collins M. (2006). Interleukin (IL)-22 and IL-17 are coexpressed by Th17 cells and cooperatively enhance expression of antimicrobial peptides. J Exp Med.

[bib15] McGeachy M.J., Chen Y., Tato C.M., Laurence A., Joyce-Shaikh B., Blumenschein W.M. (2009). The interleukin 23 receptor is essential for the terminal differentiation of interleukin 17-producing effector T helper cells in vivo. Nat Immunol.

[bib16] Mehta H., Mashiko S., Angsana J., Rubio M., Hsieh Y.M., Maari C. (2021). Differential changes in inflammatory mononuclear phagocyte and T-cell profiles within psoriatic skin during treatment with guselkumab vs. secukinumab. J Invest Dermatol.

[bib17] Navrazhina K., Renert-Yuval Y., Frew J.W., Grand D., Gonzalez J., Williams S.C. (2022). Large-scale serum analysis identifies unique systemic biomarkers in psoriasis and hidradenitis suppurativa. Br J Dermatol.

[bib18] Nussbaum L., Chen Y.L., Ogg G.S. (2021). Role of regulatory T cells in psoriasis pathogenesis and treatment. Br J Dermatol.

[bib19] Reich K., Armstrong A.W., Foley P., Song M., Wasfi Y., Randazzo B. (2017). Efficacy and safety of guselkumab, an anti-interleukin-23 monoclonal antibody, compared with adalimumab for the treatment of patients with moderate to severe psoriasis with randomized withdrawal and retreatment: results from the phase III, double-blind, placebo- and active comparator-controlled VOYAGE 2 trial. J Am Acad Dermatol.

[bib20] Reich K., Armstrong A.W., Langley R.G., Flavin S., Randazzo B., Li S. (2019). Guselkumab versus secukinumab for the treatment of moderate-to-severe psoriasis (Eclipse): results from a phase 3, randomised controlled trial. Lancet.

[bib21] Reich K., Gordon K.B., Strober B.E., Armstrong A.W., Miller M., Shen Y.K. (2021). Five-year maintenance of clinical response and health-related quality of life improvements in patients with moderate-to-severe psoriasis treated with guselkumab: results from VOYAGE 1 and VOYAGE 2. Br J Dermatol.

[bib22] Reich K., Warren R.B., Lebwohl M., Gooderham M., Strober B., Langley R.G. (2021). Bimekizumab versus secukinumab in plaque psoriasis. N Engl J Med.

[bib23] Sawyer L.M., Malottki K., Sabry-Grant C., Yasmeen N., Wright E., Sohrt A. (2019). Assessing the relative efficacy of interleukin-17 and interleukin-23 targeted treatments for moderate-to-severe plaque psoriasis: a systematic review and network meta-analysis of PASI response. PLoS One.

[bib24] Sofen H., Smith S., Matheson R.T., Leonardi C.L., Calderon C., Brodmerkel C. (2014). Guselkumab (an IL-23-specific mAb) demonstrates clinical and molecular response in patients with moderate-to-severe psoriasis. J Allergy Clin Immunol.

[bib25] Teng M.W., Bowman E.P., McElwee J.J., Smyth M.J., Casanova J.L., Cooper A.M. (2015). IL-12 and IL-23 cytokines: from discovery to targeted therapies for immune-mediated inflammatory diseases. Nat Med.

[bib26] Tian S., Krueger J.G., Li K., Jabbari A., Brodmerkel C., Lowes M.A. (2012). Meta-analysis derived (MAD) transcriptome of psoriasis defines the “core” pathogenesis of disease. PLoS One.

[bib27] Whitley S.K., Li M., Kashem S.W., Hirai T., Igyártó B.Z., Knizner K. (2022). Local IL-23 is required for proliferation and retention of skin-resident memory T_H_17 cells. Sci Immunol.

[bib28] Yao Z., Hu C., Zhu Y., Xu Z., Randazzo B., Wasfi Y. (2018). Population pharmacokinetic modeling of guselkumab, a human IgG1λ monoclonal antibody targeting IL-23, in patients with moderate to severe plaque psoriasis. J Clin Pharmacol.

[bib29] Yiu Z.Z.N., Becher G., Kirby B., Laws P., Reynolds N.J., Smith C.H. (2022). Drug survival associated with effectiveness and safety of treatment with guselkumab, ixekizumab, secukinumab, ustekinumab, and adalimumab in patients with psoriasis. JAMA Dermatol.

[bib30] Yu G., Wang L.G., Han Y., He Q.Y. (2012). clusterProfiler: an R package for comparing biological themes among gene clusters. Omics.

