# Supplementary material for: Differential Pharmacodynamic Effects on Psoriatic Biomarkers by Guselkumab Versus Secukinumab Correlate with Long-Term Efficacy: An ECLIPSE Substudy
Source: JID Innov. 2024 Jun 26;4(5):100297. doi: 10.1016/j.xjidi.2024.100297 (PMC11367549; doi:10.1016/j.xjidi.2024.100297)
Supplement: Supplementary Table [file mmc1.docx]

| Australia | Bellberry Limited  129 Glen Osmond Road,  Eastwood, South Australia, 5063  Australia | F77-AU10001  Premier Specialists  The Church, 17 Kensington Street,  Kogarah, NSW, 2217  Australia  Received drug | Site, Central: 3/31/2017 |
| --- | --- | --- | --- |
| Australia | Bellberry Limited  129 Glen Osmond Road,  Eastwood, South Australia, 5063  Australia | F77-AU10002  St George Dermatology & Skin Cancer Centre  Level 3, 22 Belgrave St ,  Kogarah, NSW, 2217  Australia  Received drug | Site, Central: 3/31/2017 |
| Australia | Melbourne Health Human Research Ethics Committee  The Royal Melbourne Hospital, Level 2 South West, 300 Grattan St,  Parkville, VIC, 3050  Australia | F77-AU10003  Westmead Hospital  Cnr Hawkesbury And Darcy Rd,  Westmead, New South Wales, 2145  Australia  Received drug | Site, Central: 4/4/2017 |
| Australia | Bellberry Limited  129 Glen Osmond Road,  Eastwood, South Australia, 5063  Australia | F77-AU10004  Skin&Cancer Foundation Inc  1/80 Drummond Street, Carlton,  Melbourne, Victoria, 3053  Australia  Received drug | Site, Central: 3/31/2017 |
| Australia | Bellberry Limited  129 Glen Osmond Road,  Eastwood, South Australia, 5063  Australia | F77-AU10005  The Skin Centre  Suite 3.07, Level 3, Pindara Specialist Suites,  29 Carrara Street,  Benowa, Queensland, 4217  Australia  Received drug | Site, Central: 3/31/2017 |
| Australia | Bellberry Limited  129 Glen Osmond Road,  Eastwood, South Australia, 5063  Australia | F77-AU10006  Fremantle Dermatology  229 High Street,  Fremantle, Western Australia, 6160  Australia  Received drug | Site, Central: 3/31/2017 |
| Australia | Bellberry Limited  129 Glen Osmond Road,  Eastwood, South Australia, 5063  Australia | F77-AU10007  Woden Dermatology  Level 1, 1 Bowes Place,  Woden, ACT, 2606  Australia  Received drug | Site, Central: 3/31/2017 |
| Australia | Bellberry Limited  129 Glen Osmond Road,  Eastwood, South Australia, 5063  Australia | F77-AU10008  Clinical Trials SA Pty Ltd  230, St Bernards Road,  Hectorville, South Australia, 5073  Australia  Received drug | Site, Central: 3/31/2017 |
| Australia | Bellberry Limited  129 Glen Osmond Road,  Eastwood, South Australia, 5063  Australia | F77-AU10009  Sinclair Dermatology  2 Wellington Parade,  East Melbourne, 3002  Australia  Received drug | Site, Central: 3/31/2017 |
| Australia | Bellberry Limited  129 Glen Osmond Road,  Eastwood, South Australia, 5063  Australia | F77-AU10010  Veracity Clinical Research  Suite 18, Level 1, 250 Ipswich Road,  Woolloongabba, Queensland, 4102  Australia  Received drug | Site, Central: 3/31/2017 |
| Australia | Melbourne Health Human Research Ethics Committee  The Royal Melbourne Hospital, Level 2 South West, 300 Grattan St,  Parkville, VIC, 3050  Australia | F77-AU10011  Royal Melbourne Hospital  Grattan Street, 8th Floor, Room W8003,  Parkville, Victoria, 3050  Australia  Received drug | Site, Central: 4/4/2017 |
| Canada | Veritas IRB Inc.  8555 Transcanada Hwy, Suite 201,  Montreal, Quebec, H4S 1Z6  Canada | F77-CA10001  CCA Medical Research Corporation  95 Bayly Street West,Suite G-02, Immunology,  Ajax, L1S7K8  Canada  Received drug | Site, Central: 4/19/2017 |
| Canada | Veritas IRB Inc.  8555 Transcanada Hwy, Suite 201,  Montreal, Quebec, H4S 1Z6  Canada | F77-CA10002  Skin Centre for Dermatology  775 Monaghan Road,  Peterborough, Ontario, K9J 5K2  Canada  Received drug | Site, Central: 4/12/2017 |
| Canada | Veritas IRB Inc.  8555 Transcanada Hwy, Suite 201,  Montreal, Quebec, H4S 1Z6  Canada | F77-CA10003  Guenther Dermatology Research Centre  835 Richmond Street,  London, Ontario, N6A 3H7  Canada  Received drug | Site, Central: 4/27/2017 |
| Canada | Veritas IRB Inc.  8555 Transcanada Hwy, Suite 201,  Montreal, Quebec, H4S 1Z6  Canada | F77-CA10004  DermEdge Research  333 Lakeshore Road West, Suite 102, Immunology,  Mississauga, L5H 1G9  Canada  Received drug | Site, Central: 4/12/2017 |
| Canada | Veritas IRB Inc.  8555 Transcanada Hwy, Suite 201,  Montreal, Quebec, H4S 1Z6  Canada | F77-CA10005  K. Papp Clinical Research  135 Union Street East, Immunology,  Waterloo, N2J 1C4  Canada  Received drug | Site, Central: 4/11/2017 |
| Canada | Veritas IRB Inc.  8555 Transcanada Hwy, Suite 201,  Montreal, Quebec, H4S 1Z6  Canada | F77-CA10006  XLR8 Medical Research  2425 Tecumseh Road East, Suite 210, Dermatology,  Windsor, N8W 1E6  Canada  Received drug | Site, Central: 4/11/2017 |
| Canada | Veritas IRB Inc.  8555 Transcanada Hwy, Suite 201,  Montreal, Quebec, H4S 1Z6  Canada | F77-CA10007  Dermatrials Research  25 Charlton Avenue East, Suite 707,  Hamilton, ON, L8N 1Y2  Canada  Received drug | Site, Central: 4/14/2017 |
| Canada | Health Research Ethics Board of Alberta Clinical Trials Committee (HREBA)  1500, 10104 - 103 Avenue NW  Edmonton, Alberta, T5J 0H8  Canada | F77-CA10008  Stratica Medical  10140 117 Street NW, Suite 200, Dermatology,  Edmonton, T5K 1X3  Canada  Received drug | Site, Local: 4/28/2017 |
| Canada | Nova Scotia Health Authority Research Ethics Board (NSHA REB)  Centre for Clinical Research, Room 118 5790 University Avenue,  Halifax, Nova Scotia, B3H 1V7  Canada | F77-CA10009  Eastern Canada Research Associates  6054 Coburg Road, Immunology,  Halifax, B3H 1Z2  Canada  Received drug | Site, Local: 05/05/2017 |
| Canada | Veritas IRB Inc.  8555 Transcanada Hwy, Suite 201,  Montreal, Quebec, H4S 1Z6  Canada | F77-CA10010  Innovaderm Research  1851 Sherbrooke Street East, Suite 502, Immunology,  Montreal, H2K4L5  Canada  Received drug | Site, Central: 4/27/2017 |
| Canada | Veritas IRB Inc.  8555 Transcanada Hwy, Suite 201,  Montreal, Quebec, H4S 1Z6  Canada | F77-CA10011  North Bay Dermatology Centre  500 Cassells Street,  North Bay, Ontario, P1B 3Z7  Canada  Received drug | Site, Central: 4/11/2017 |
| Canada | Veritas IRB Inc.  8555 Transcanada Hwy, Suite 201,  Montreal, Quebec, H4S 1Z6  Canada | F77-CA10013  Dr. Chih-ho Hong Medical  15300 105 Avenue,Suite 20, Dermatology,  Surrey, V3R 6A7  Canada  Received drug | Site, Central: 4/11/2017 |
| Canada | Veritas IRB Inc.  8555 Transcanada Hwy, Suite 201,  Montreal, Quebec, H4S 1Z6  Canada | F77-CA10014  Centre Dermatologique  150-2880 Chemin des Quatre-Bourgeois, Immunology,  Quebec, G1V 4X7  Canada  Received drug | Site, Central: 4/27/2017 |
| Canada | Veritas IRB Inc.  8555 Transcanada Hwy, Suite 201,  Montreal, Quebec, H4S 1Z6  Canada | F77-CA10016  Toronto Research Centre  4256 Bathrust Street, Suite 301,  Toronto, ON, M3H5Y8  Canada  Received drug | Site, Central: 4/12/2017 |
| Czechia | Lokalni Eticka komise FN Ostrava  17. listopadu, 1790/5  Ostrava-Poruba, 70852  Czechia | F77-CZ10001  Fakultni nemocnice Ostrava  17. listopadu 1790/5, Kozni oddeleni,  Ostrava- Poruba, 708 52  Czechia  Received drug | Site, Central: 3/29/2017  Site, Local: 4/27/2017 |
| Czechia | Eticka komise pro multicentricke klinicke hodnoceni Fakultni nemocnice v Motole  V Uvalu 84,  Praha 5 , 150 06  Czechia | F77-CZ10001  Fakultni nemocnice Ostrava  17. listopadu 1790/5, Kozni oddeleni,  Ostrava- Poruba, 708 52  Czechia  Received drug | Site, Central: 3/29/2017  Site, Local: 4/27/2017 |
| Czechia | Eticka komise Nemocnice Novy Jicin  Purkynova 2138/16,  Nový Jicin, 74101  Czechia | F77-CZ10003  Nemocnice Novy Jicin a.s.  Purkynova 2138/16, Kozni oddeleni,  Novy Jicin, 741 01  Czechia  Received drug | Site, Central: 3/29/2017  Site, Local: 3/29/2017 |
| Czechia | Eticka komise pro multicentricke klinicke hodnoceni Fakultni nemocnice v Motole  V Uvalu 84,  Praha 5, 150 06  Czechia | F77-CZ10003  Nemocnice Novy Jicin a.s.  Purkynova 2138/16, Kozni oddeleni,  Novy Jicin, 741 01  Czechia  Received drug | Site, Central: 3/29/2017  Site, Local: 3/29/2017 |
| Czechia | Eticka komise Fakultni nemocnice Hradec Kralove  Sokolska 581,  Hradec Kralove, 50005  Czechia | F77-CZ10005  Dermatologicka ambulance  Hranicni 2118/9  Svitavy, 568 02  Czechia  Received drug | Site, Central: 3/29/2017  Site, Local: 4/6/2017 |
| Czechia | Eticka komise pro multicentricke klinicke hodnoceni Fakultni nemocnice v Motole  V Uvalu 84,  Praha 5, 150 06  Czechia | F77-CZ10005  Dermatologicka ambulance  Hranicni 2118/9  Svitavy, 568 02  Czechia  Received drug | Site, Central: 3/29/2017  Site, Local: 4/6/2017 |
| Czechia | Eticka komise pro multicentricke klinicke hodnoceni Fakultni nemocnice v Motole  V Uvalu 84,  Praha 5, 150 06  Czechia | F77-CZ10006  Kozni ambulance Kutna Hora, s.r.o.  Kourimska 915  Kutna Hora, 284 01  Czechia  Received drug | Site, Central: 3/29/2017  Site, Local: 4/6/2017 |
| Czechia | Eticka komise Fakultni nemocnice Hradec Kralove  Eticka komise Fakultni nemocnice Hradec Kralove, Sokolska 581,  Hradec Kralove, 50005  Czechia | F77-CZ10006  Kozni ambulance Kutna Hora, s.r.o.  Kourimska 915  Kutna Hora, 284 01  Czechia  Received drug | Site, Central: 3/29/2017  Site, Local: 4/6/2017 |
| Czechia | Eticka komise pro multicentricke klinicke hodnoceni Fakultni nemocnice v Motole  V Uvalu 84,  Praha 5, 150 06  Czechia | F77-CZ10007  Fakultni nemocnice Kralovske Vinohrady  Srobarova 1150/50, Dermatology  Praha, 775 20  Czechia  Received drug | Site, Central: 3/29/2017  Site, Local: 4/5/2017 |
| Czechia | Eticka komise Fakultni nemocnice Kralovske Vinohrady  Srobarova 50,  Praha 10, 10034  Czechia | F77-CZ10007  Fakultni nemocnice Kralovske Vinohrady  Srobarova 1150/50, Dermatology  Praha, 775 20  Czechia  Received drug | Site, Central: 3/29/2017  Site, Local: 4/5/2017 |
| Czechia | Eticka komise Krajska zdravotni a.s.- Masarykova nem. Usti n. Labem  Socialni Pece 12A, N/A, N/A,  Usti Nad Labem N/a, 401 13  Czechia | F77-CZ10009  Masarykova nemocnice v Usti nad Labem  Socialni Pece 3316 /12A, Dermatology  Usti Nad Labem  Czechia  Received drug | Site, Central: 3/29/2017  Site, Local: 3/15/2017 |
| Czechia | Eticka komise pro multicentricke klinicke hodnoceni Fakultni nemocnice v Motole  V Uvalu 84,  Praha 5, 150 06  Czechia | F77-CZ10009  Masarykova nemocnice v Usti nad Labem  Socialni Pece 3316 /12A, Dermatology  Usti Nad Labem  Czechia  Received drug | Site, Central: 3/29/2017  Site, Local: 3/15/2017 |
| Czechia | Eticka komise pro multicentricke klinicke hodnoceni Fakultni nemocnice v Motole  V Uvalu 84,  Praha 5, 150 06  Czechia | F77-CZ10010  DERMAMEDICA s.r.o.  Prazska 1995, Immunology  Nachod, 547 01  Czechia  Received drug | Site, Central: 3/29/2017  Site, Local: 4/6/2017 |
| Czechia | Eticka komise Fakultni nemocnice Hradec Kralove  Sokolska 581,  Hradec Kralove, 50005  Czechia | F77-CZ10010  DERMAMEDICA s.r.o.  Prazska 1995, Immunology  Nachod, 547 01  Czechia  Received drug | Site, Central: 3/29/2017  Site, Local: 4/6/2017 |
| Czechia | Eticka komise Nemocnice Jihlava  Vrchlickeho 59,  Jihlava, 586 33  Czechia | F77-CZ10012  Nemocnice Jihlava  Vrchlickeho 59  Jihlava, 586 33  Czechia  Received drug | Site, Central: 3/29/2017  Site, Local: 4/16/2017 |
| Czechia | Eticka komise pro multicentricke klinicke hodnoceni Fakultni nemocnice v Motole  V Uvalu 84,  Praha 5, 150 06  Czechia | F77-CZ10012  Nemocnice Jihlava  Vrchlickeho 59  Jihlava, 586 33  Czechia  Received drug | Site, Central: 3/29/2017  Site, Local: 4/16/2017 |
| France | Comité de Protection des Personnes Ouest VI  CHRU La Cavale Blanche, Boulevard Tanguy Prigent,  Brest, 29200  France | F77-FR10001  Le Bateau Blanc  26 Chemin de Paradis, Service de Dermatologie,  Martigues, 13500  France  Received drug | Region: 3/21/2017 |
| France | Comité de Protection des Personnes Ouest VI  CHRU La Cavale Blanche, Boulevard Tanguy Prigent,  Brest, 29200  France | F77-FR10002  Hopital Larrey CHU de Toulouse  24, chemin de Pouvourville,  Toulouse, 31000  France  Received drug | Region: 3/21/2017 |
| France | Comité de Protection des Personnes Ouest VI  CHRU La Cavale Blanche, Boulevard Tanguy Prigent,  Brest, 29200  France | F77-FR10003  Hopital Charles Nicolle  1 rue de Germont, Cour Germont Porte 12, Service de Dermatologie,  Rouen, 76031  France  Received drug | Region: 3/21/2017 |
| France | Comité de Protection des Personnes Ouest VI  CHRU La Cavale Blanche, Boulevard Tanguy Prigent,  Brest, 29200  France | F77-FR10004  CHU de Nice Hopital de l Archet  151 route de St Antoine de Ginestiere, Service de Dermatologie,  Nice, 06200  France  Received drug | Region: 3/21/2017 |
| France | Comité de Protection des Personnes Ouest VI  CHRU La Cavale Blanche, Boulevard Tanguy Prigent,  Brest, 29200  France | F77-FR10005  CHU Bordeaux - Hopital St Andre  1 Rue Jean Burguet, Service de Dermatologie,  Bordeaux, 33000  France  Received drug | Region: 3/21/2017 |
| France | Comité de Protection des Personnes Ouest VI  CHRU La Cavale Blanche, Boulevard Tanguy Prigent,  Brest, 29200  France | F77-FR10009  ICH Hopital A. Morvan  2 Avenue Marechal Foch, Service de Dermatologie et venereologie,  Brest, 29200  France  Received drug | Region: 3/21/2017 |
| France | Comité de Protection des Personnes Ouest VI  CHRU La Cavale Blanche, Boulevard Tanguy Prigent,  Brest, 29200  France | F77-FR10013  CHU Nantes - Hotel Dieu  1 place Alexis-Ricordeau, 7e etage aile nord, Service de Dermatologie,  Nantes, 44093  France  Received drug | Region: 3/21/2017 |
| France | Comité de Protection des Personnes Ouest VI  CHRU La Cavale Blanche, Boulevard Tanguy Prigent,  Brest, 29200  France | F77-FR10014  Groupe Hospitalier La Rochelle - Re - Aunis  Rue du docteur Schweitzer, Service de Dermatologie,  La Rochelle, 17019  France  Received drug | Region: 3/21/2017 |
| Germany | Landesamt für Gesundheit und Soziales Berlin Geschäftsstelle der Ethik-Kommission des Landes Berlin  Fehrbelliner Platz 1,  Berlin, 10707  Germany | F77-DE10001  Charite Universitatsmedizin Berlin, Campus Mitte (CCM) Allergie Center  Chariteplatz 1, Klinik fur Dermatologie, Venerologie und Allergologie; Psoriasis Studienzentrum,  Berlin, 10117  Germany  Received drug | Region: 5/10/2017  Site, Local: 5/10/2017 |
| Germany | Ethik-Kommission an der Medizinischen Fakultät der Eberhard-Karls-Universität und am Universitätsklinikum Tübingen  Gartenstraße 47,  Tübingen, 72074  Germany | F77-DE10001  Charite Universitatsmedizin Berlin, Campus Mitte (CCM) Allergie Center  Chariteplatz 1, Klinik fur Dermatologie, Venerologie und Allergologie; Psoriasis Studienzentrum,  Berlin, 10117  Germany  Received drug | Region: 5/10/2017  Site, Local: 5/10/2017 |
| Germany | Ethik-Kommission an der Medizinischen Fakultät der Eberhard-Karls-Universität und am Universitätsklinikum Tübingen  Gartenstraße 47,  Tübingen, 72074  Germany | F77-DE10002  Technische Universitaet Muenchen  Biedersteiner Str. 29,  Muenchen, 80802  Germany  Received drug | Region: 5/10/2017  Site, Local: 5/10/2017 |
| Germany | Ethikkommission der Medizinischen Fakultät der Technischen Universität München  Ismaninger Str. 22,  München, 81675  Germany | F77-DE10002  Technische Universitaet Muenchen  Biedersteiner Str. 29,  Muenchen, 80802  Germany  Received drug | Region: 5/10/2017  Site, Local: 5/10/2017 |
| Germany | Ethik-Kommission des Fachbereichs Medizin der Johann Wolfgang Goethe-Universität  Theodor-Stern-Kai 7, Haus 1, 2. OG, Zi. 222/223,  Frankfurt, Hesse, 60590  Germany | F77-DE10003  Universitatsklinikum Frankfurt  Theodor-Stern-Kai 7, Klinik fur Dermatologie, Venerologie und Allergologie,  Frankfurt am Main, 60590  Germany  Received drug | Region: 5/10/2017  Site, Local: 5/10/2017 |
| Germany | Ethik-Kommission an der Medizinischen Fakultät der Eberhard-Karls-Universität und am Universitätsklinikum Tübingen  Gartenstraße 47,  Tübingen, 72074  Germany | F77-DE10003  Universitatsklinikum Frankfurt  Theodor-Stern-Kai 7, Klinik fur Dermatologie, Venerologie und Allergologie,  Frankfurt am Main, 60590  Germany  Received drug | Region: 5/10/2017  Site, Local: 5/10/2017 |
| Germany | Ethik-Kommission an der medizinischen Fakultät der Rheinischen Friedrich- Wilhelms-Universität Bonn - Biomedizinsches Zentrum  Sigmund-Freud-Str. 25,  Bonn, 53105  Germany | F77-DE10004  Universitatsklinikum Bonn  Siegmund-Freud-Str. 23, Klinik und Poliklinik fur Dermatologie und Allergologie,  Bonn, 53105  Germany  Received drug | Region: 5/10/2017  Site, Local: 5/10/2017 |
| Germany | Ethik-Kommission an der Medizinischen Fakultät der Eberhard-Karls-Universität und am Universitätsklinikum Tübingen  Gartenstraße 47,  Tübingen, 72074  Germany | F77-DE10004  Universitatsklinikum Bonn  Siegmund-Freud-Str. 23, Klinik und Poliklinik fur Dermatologie und Allergologie,  Bonn, 53105  Germany  Received drug | Region: 5/10/2017  Site, Local: 5/10/2017 |
| Germany | Ethik-Kommission der Medizinischen Fakultät der Christian-Albrechts-Universität zu Kiel  Arnold-Heller-Str. 3, Haus 9,  Kiel, 24105  Germany | F77-DE10005  Universitatsklinikum Schleswig-Holstein - Kiel  Arnold-Heller 3, Haus 19, Abt. Dermatologie, Venerologie und Allergologie,  Kiel, 24105  Germany  Received drug | Region: 5/10/2017  Site, Local: 5/10/2017 |
| Germany | Ethik-Kommission an der Medizinischen Fakultät der Eberhard-Karls-Universität und am Universitätsklinikum Tübingen  Gartenstraße 47,  Tübingen, 72074  Germany | F77-DE10005  Universitatsklinikum Schleswig-Holstein - Kiel  Arnold-Heller 3, Haus 19, Abt. Dermatologie, Venerologie und Allergologie,  Kiel, 24105  Germany  Received drug | Region: 5/10/2017  Site, Local: 5/10/2017 |
| Germany | Ethik-Kommission an der Medizinischen Fakultät der Eberhard-Karls-Universität und am Universitätsklinikum Tübingen  Gartenstraße 47,  Tübingen, 72074  Germany | F77-DE10006  Universitaetsklinik Tuebingen  Liebermeisterstr 25,  Tuebingen, 72076  Germany  Received drug | Region: 5/10/2017 |
| Germany | Ethikkommission der Ärztekammer Hamburg; Körperschaft des öffentlichen Rechts  Weidestr. 122b,  Hamburg, HH, 22083  Germany | F77-DE10007  SCIderm GmbH  Drehbahn 1-3,  Hamburg, 20354  Germany  Received drug | Region: 5/10/2017  Site, Local: 5/10/2017 |
| Germany | Ethik-Kommission an der Medizinischen Fakultät der Eberhard-Karls-Universität und am Universitätsklinikum Tübingen  Gartenstraße 47,  Tübingen, 72074  Germany | F77-DE10007  SCIderm GmbH  Drehbahn 1-3,  Hamburg, 20354  Germany  Received drug | Region: 5/10/2017  Site, Local: 5/10/2017 |
| Germany | Ethik-Kommission an der Medizinischen Fakultät der Eberhard-Karls-Universität und am Universitätsklinikum Tübingen  Gartenstraße 47,  Tübingen, 72074  Germany | F77-DE10008  Hautarztpraxis  Am Bahnhof 1, Gemeinschaftspraxis Dres. Scholz/Sebastian/Schilling,  Mahlow, 15831  Germany  Received drug | Region: 5/10/2017  Site, Local: 5/10/2017 |
| Germany | Ethik-Kommission der Landesärztekammer Brandenburg  Dreifertstr. 12,  Cottbus, Brandenburg, 03044  Germany | F77-DE10008  Hautarztpraxis  Am Bahnhof 1, Gemeinschaftspraxis Dres. Scholz/Sebastian/Schilling,  Mahlow, 15831  Germany  Received drug | Region: 5/10/2017  Site, Local: 5/10/2017 |
| Germany | Ethik-Komission der Universität zu Lübeck  Ratzeburger Allee 160,  Lubeck, 23538  Germany | F77-DE10009  Universitaetsklinik Luebeck  Ratzeburger Allee 160,  Luebeck, 23538  Germany  Received drug | Region: 5/10/2017  Site, Local: 5/10/2017 |
| Germany | Ethik-Kommission an der Medizinischen Fakultät der Eberhard-Karls-Universität und am Universitätsklinikum Tübingen  Gartenstraße 47,  Tübingen, 72074  Germany | F77-DE10009  Universitaetsklinik Luebeck  Ratzeburger Allee 160,  Luebeck, 23538  Germany  Received drug | Region: 5/10/2017  Site, Local: 5/10/2017 |
| Germany | Ethik-Kommission der Medizinischen Fakultät der Universität Duisburg-Essen  Robert-Koch-Str. 9-11,  Essen, 45122  Germany | F77-DE10010  Universitatsklinikum Essen  Hufelandstrasse 55, Dermatologie,  Essen, 45122  Germany  Received drug | Region: 5/10/2017  Site, Local: 5/10/2017 |
| Germany | Ethik-Kommission an der Medizinischen Fakultät der Eberhard-Karls-Universität und am Universitätsklinikum Tübingen  Gartenstraße 47,  Tübingen, 72074  Germany | F77-DE10010  Universitatsklinikum Essen  Hufelandstrasse 55, Dermatologie,  Essen, 45122  Germany  Received drug | Region: 5/10/2017  Site, Local: 5/10/2017 |
| Germany | Ethik-Kommission an der Medizinischen Fakultät der Eberhard-Karls-Universität und am Universitätsklinikum Tübingen  Gartenstraße 47,  Tübingen, 72074  Germany | F77-DE10011  Klinische Forschung Dresden GmbH  Prager Str. 10,  Dresden, 01069  Germany  Received drug | Region: 5/10/2017  Site, Local: 5/10/2017 |
| Germany | Ethikkommission der Sächsischen Landesärztekammer  Schützenhöhe 16-18,  Dresden, 01099  Germany | F77-DE10011  Klinische Forschung Dresden GmbH  Prager Str. 10,  Dresden, 01069  Germany  Received drug | Region: 5/10/2017  Site, Local: 5/10/2017 |
| Germany | Ethikkommission der Ärztekammer Hamburg; Körperschaft des öffentlichen Rechts  Weidestr. 122b, Hamburg, HH, 22083  Germany | F77-DE10012  Universitaetsklinik Hamburg-Eppendorf  Martinistr. 52,  Hamburg, 20246  Germany  Received drug | Region: 5/10/2017  Site, Local: 5/10/2017 |
| Germany | Ethik-Kommission an der Medizinischen Fakultät der Eberhard-Karls-Universität und am Universitätsklinikum Tübingen  Gartenstraße 47,  Tübingen, 72074  Germany | F77-DE10012  Universitaetsklinik Hamburg-Eppendorf  Martinistr. 52,  Hamburg, 20246  Germany  Received drug | Region: 5/10/2017  Site, Local: 5/10/2017 |
| Germany | Ethikkommission Technische Universität Dresden  Fetscherstraße 74,  Dresden, Saxony, 01307  Germany | F77-DE10013  University Hospital Dresden  Fetscherstr. 74, Klinik und Poliklinik fur Dermatologie,  Dresden, 01307  Germany  Received drug | Region: 5/10/2017  Site, Local: 5/10/2017 |
| Germany | Ethik-Kommission an der Medizinischen Fakultät der Eberhard-Karls-Universität und am Universitätsklinikum Tübingen  Gartenstraße 47,  Tübingen, 72074  Germany | F77-DE10013  University Hospital Dresden  Fetscherstr. 74, Klinik und Poliklinik fur Dermatologie,  Dresden, 01307  Germany  Received drug | Region: 5/10/2017  Site, Local: 5/10/2017 |
| Germany | Ethik-Kommission der Ärztekammer Westfalen-Lippe und der Medizinischen Fakultät der Westfälischen Wilhelms-Universität Münster  Gartenstr. 210-214,  Münster, Nordrhein-Westfalen, 48147  Germany | F77-DE10014  Centrovital  Annenstr. 151,  Witten, 58453  Germany  Received drug | Region: 5/10/2017  Site, Local: 5/10/2017 |
| Germany | Ethik-Kommission an der Medizinischen Fakultät der Eberhard-Karls-Universität und am Universitätsklinikum Tübingen  Gartenstraße 47,  Tübingen, 72074  Germany | F77-DE10014  Centrovital  Annenstr. 151,  Witten, , 58453  Germany  Received drug | Region: 5/10/2017  Site, Local: 5/10/2017 |
| Germany | Landesamt für Gesundheit und Soziales Berlin Geschäftsstelle der Ethik-Kommission des Landes Berlin  Fehrbelliner Platz 1,  Berlin, 10707  Germany | F77-DE10015  ISA GmbH  Rankestrasse 34,  Berlin, 10789  Germany  Received drug | Region: 5/10/2017  Site, Local: 5/10/2017 |
| Germany | Ethik-Kommission an der Medizinischen Fakultät der Eberhard-Karls-Universität und am Universitätsklinikum Tübingen  Gartenstraße 47,  Tübingen, 72074  Germany | F77-DE10015  ISA GmbH  Rankestrasse 34,  Berlin, 10789  Germany  Received drug | Region: 5/10/2017  Site, Local: 5/10/2017 |
| Germany | Ethikkommission der Ärztekammer Hamburg; Körperschaft des öffentlichen Rechts  Weidestr. 122b,  Hamburg, HH, 22083  Germany | F77-DE10016  MensingDerma research GmbH  Heegbarg 4,  Hamburg, 22391  Germany  Received drug | Region: 5/10/2017  Site, Local: 5/10/2017 |
| Germany | Ethik-Kommission an der Medizinischen Fakultät der Eberhard-Karls-Universität und am Universitätsklinikum Tübingen  Gartenstraße 47,  Tübingen, 72074  Germany | F77-DE10016  MensingDerma research GmbH  Heegbarg 4,  Hamburg, 22391  Germany  Received drug | Region: 5/10/2017  Site, Local: 5/10/2017 |
| Germany | Ethik-Kommission der Ärztekammer Westfalen-Lippe und der Medizinischen Fakultät der Westfälischen Wilhelms-Universität Münster  Gartenstr. 210-214,  Münster, Nordrhein-Westfalen, 48147  Germany | F77-DE10017  Universitaetsklinikum Muenster  Von-Esmarch-Strasse 58,  Muenster, 48149  Germany  Received drug | Region: 5/10/2017  Site, Local: 5/10/2017 |
| Germany | Ethik-Kommission an der Medizinischen Fakultät der Eberhard-Karls-Universität und am Universitätsklinikum Tübingen  Gartenstraße 47,  Tübingen, 72074  Germany | F77-DE10017  Universitaetsklinikum Muenster  Von-Esmarch-Strasse 58,  Muenster, 48149  Germany  Received drug | Region: 5/10/2017  Site, Local: 5/10/2017 |
| Hungary | Central Ethics Committee Medical Research Council Ethics Committee for Clinical Pharmacology  Arany János Utca 6-8,  Budapest, 1051  Hungary | F77-HU10001  Markusovszky Egyetemi Oktatokorhaz  Markusovszky u. 5,  Szombathely, 9700  Hungary  Received drug | Region: 3/30/2017 |
| Hungary | Central Ethics Committee Medical Research Council Ethics Committee for Clinical Pharmacology  Arany János Utca 6-8,  Budapest, 1051  Hungary | F77-HU10002  Medmare Egeszsegugyi Es Szolgaltato Bt.  Jozsef Attila u. 17,  Veszprem, 8200  Hungary  Received drug | Region: 3/30/2017 |
| Hungary | Central Ethics Committee Medical Research Council Ethics Committee for Clinical Pharmacology  Arany János Utca 6-8,  Budapest, 1051  Hungary | F77-HU10003  Bacs-kiskun Megyei Korhaz  Nylri ut 38.,  Kecskemet, 6000  Hungary  Received drug | Region: 3/30/2017 |
| Hungary | Central Ethics Committee Medical Research Council Ethics Committee for Clinical Pharmacology  Arany János Utca 6-8,  Budapest, 1051  Hungary | F77-HU10004  Somogy Megyei Kaposi Mor Oktatokorhaz  Tallian Gy. u. 20-32.,  Kaposvar, 7400  Hungary  Received drug | Region: 3/30/2017 |
| Hungary | Central Ethics Committee Medical Research Council Ethics Committee for Clinical Pharmacology  Arany János Utca 6-8,  Budapest, 1051  Hungary | F77-HU10005  Semmelweis Egyetem  Maria u. 41,  Budapest, 1085  Hungary  Received drug | Region: 3/30/2017 |
| Hungary | Central Ethics Committee Medical Research Council Ethics Committee for Clinical Pharmacology  Arany János Utca 6-8,  Budapest, 1051  Hungary | F77-HU10006  Szegedi Tudomanyegyetem  Koranyi Fasor 6,  Szeged, 6720  Hungary  Received drug | Region: 3/30/2017 |
| Hungary | Central Ethics Committee Medical Research Council Ethics Committee for Clinical Pharmacology  Arany János Utca 6-8,  Budapest, 1051  Hungary | F77-HU10007  Debreceni Egyetem Klinikai Kozpont  Nagyerdei krt. 98.,  Debrecen, 4032  Hungary  Received drug | Region: 3/30/2017 |
| Hungary | Central Ethics Committee Medical Research Council Ethics Committee for Clinical Pharmacology  Arany János Utca 6-8,  Budapest, 1051  Hungary | F77-HU10008  Pecsi Tudomanyegyetem  Akac u. 1.,  Pecs, 7632  Hungary  Received drug | Region: 3/30/2017 |
| Hungary | Central Ethics Committee Medical Research Council Ethics Committee for Clinical Pharmacology  Arany János Utca 6-8,  Budapest, 1051  Hungary | F77-HU10009  Borsod-Abauj-Zemplen Megyei Korhaz es Egyetemi Oktato Korhaz  Csabai kapu 9-11., Borgyogyaszati Osztaly,  Miskolc, 3529  Hungary  Received drug | Region: 3/30/2017 |
| Poland | Komisja Bioetyczna przy Dolnoslaskiej Izbie Lekarskiej we Wroclawiu  Kazimierza Wielkiego 45,  Wroclaw, 50-077  Poland | F77-PL10001  Lubelskie Centrum Diagnostyczne  ul. Drewniana 61  Swidnik, 21-040  Poland  Received drug | Site, Central: 4/12/2017 |
| Poland | Komisja Bioetyczna przy Dolnoslaskiej Izbie Lekarskiej we Wroclawiu  Kazimierza Wielkiego 45,  Wroclaw, 50-077  Poland | F77-PL10002  Centrum Terapii Wspolczesnej  ul. Doktora Stefana Kopcinskiego 21  Lodz, 90-242  Poland  Received drug | Site, Central: 4/12/2017 |
| Poland | Komisja Bioetyczna przy Dolnoslaskiej Izbie Lekarskiej we Wroclawiu  Kazimierza Wielkiego 45,  Wroclaw, 50-077  Poland | F77-PL10003  DermMedica Sp. z o.o.  ul. Zakrzowska 19A  Wroclaw, 51-318  Poland  Received drug | Site, Central: 4/12/2017 |
| Poland | Komisja Bioetyczna przy Dolnoslaskiej Izbie Lekarskiej we Wroclawiu  Kazimierza Wielkiego 45,  Wroclaw, 50-077  Poland | F77-PL10004  NZOZ Specjalistyczna Przychodnia Dermatologiczna SepcDerm  ul. Prezydenta Ryszarda Kaczorowskiego 7 lok. 50 U  Bialystok, 15-375  Poland  Received drug | Site, Central: 4/12/2017 |
| Poland | Komisja Bioetyczna przy Dolnoslaskiej Izbie Lekarskiej we Wroclawiu  Kazimierza Wielkiego 45,  Wroclaw, 50-077  Poland | F77-PL10005  WROMEDICA I. Bielicka, A. Strzalkowska s.c.  ul. Dabrowskiego 77a  Poznan, 60-529  Poland  Received drug | Site, Central: 4/12/2017 |
| Poland | Komisja Bioetyczna przy Dolnoslaskiej Izbie Lekarskiej we Wroclawiu  Kazimierza Wielkiego 45,  Wroclaw, 50-077  Poland | F77-PL10006  WROMEDICA I. Bielicka, A. Strzalkowska s.c.  ul. Mickiewicza 91  Wroclaw, 51-685  Poland  Received drug | Site, Central: 4/12/2017 |
| Poland | Komisja Bioetyczna przy Dolnoslaskiej Izbie Lekarskiej we Wroclawiu  Kazimierza Wielkiego 45,  Wroclaw, 50-077  Poland | F77-PL10007  Centrum Kliniczno Badawcze  ul. Studzienna 35-36/A  Elblag, 82-300  Poland  Received drug | Site, Central: 4/12/2017 |
| Poland | Komisja Bioetyczna przy Dolnoslaskiej Izbie Lekarskiej we Wroclawiu  Kazimierza Wielkiego 45,  Wroclaw, 50-077  Poland | F77-PL10008  High-Med Przychodnia Specjalistyczna  ul. Kasprowicza 27/2  Warszawa, 01-817  Poland  Received drug | Site, Central: 4/12/2017 |
| Poland | Komisja Bioetyczna przy Dolnoslaskiej Izbie Lekarskiej we Wroclawiu  Kazimierza Wielkiego 45,  Wroclaw, 50-077  Poland | F77-PL10009  Szpital Uniwersytecki nr 1 im. Dr A. Jurasza  ul. Sklodowskiej-Curie 9, Klinika Dermatologii, Chorob Przenoszonych Droga Plciowa i Immunodermatologii  Bydgoszcz, 85-094  Poland  Received drug | Site, Central: 4/12/2017 |
| Poland | Komisja Bioetyczna przy Dolnoslaskiej Izbie Lekarskiej we Wroclawiu  Kazimierza Wielkiego 45,  Wroclaw, 50-077  Poland | F77-PL10010  Copernicus Podmiot Leczniczy Sp. z o.o  ul .Powstancow Warszawskich 1-2,Oddzial Dermatologii  Gdansk, 80-298  Poland  Received drug | Site, Central: 4/12/2017 |
| Poland | Komisja Bioetyczna przy Dolnoslaskiej Izbie Lekarskiej we Wroclawiu  Kazimierza Wielkiego 45,  Wroclaw, 50-077  Poland | F77-PL10011  CRC Sp. z o.o.  ul. Poznanska 3/31  Poznan, 60-848  Poland  Received drug | Site, Central: 4/12/2017 |
| Poland | Komisja Bioetyczna przy Dolnoslaskiej Izbie Lekarskiej we Wroclawiu  Kazimierza Wielkiego 45,  Wroclaw, 50-077  Poland | F77-PL10012  NZOZ Osteo-Medic S.C. Artur Racewicz i Jerzy Supronik  ul. Wiejska 81  Bialystok, 15-351  Poland  Received drug | Site, Central: 4/12/2017 |
| Poland | Komisja Bioetyczna przy Dolnoslaskiej Izbie Lekarskiej we Wroclawiu  Kazimierza Wielkiego 45,  Wroclaw, 50-077  Poland | F77-PL10013  Dermed Centrum Medyczne Sp. z o.o  ul. Piotrkowska 48  Lodz, 90-265  Poland  Received drug | Site, Central: 4/12/2017 |
| Poland | Komisja Bioetyczna przy Dolnoslaskiej Izbie Lekarskiej we Wroclawiu  Kazimierza Wielkiego 45,  Wroclaw, 50-077  Poland | F77-PL10014  NZOZ Poradnia Dermatologiczno-Wenerologiczna Mediderm  ul. Krasinskiego 4/4A  Torun, 87-100  Poland  Received drug | Site, Central: 4/12/2017 |
| Poland | Komisja Bioetyczna przy Dolnoslaskiej Izbie Lekarskiej we Wroclawiu  Kazimierza Wielkiego 45,  Wroclaw, 50-077  Poland | F77-PL10015  Wojskowy Instytut Medyczny  ul. Szaserow 128, Klinika Dermatologiczna  Warszawa, 04-141  Poland  Received drug | Site, Central: 4/12/2017 |
| Poland | Komisja Bioetyczna przy Dolnoslaskiej Izbie Lekarskiej we Wroclawiu  Kazimierza Wielkiego 45,  Wroclaw, 50-077  Poland | F77-PL10016  Malopolskie Centrum Medyczne  ul Rejtana 2  Krakow, 30-510  Poland  Received drug | Site, Central: 4/12/2017 |
| Spain | Secretaria i Coordinació Comitè Ètic d'Investigació Clínica del Hospital Germans Trias i Pujol  Carretera Del Canyet S/n,  Badalona,08916  Spain | F77-ES10001  HOSP. UNIV. DE CRUCES  Dermatology Department  Plaza de Cruces, s/n  Barakaldo, Spain, 48903  Spain  Received drug | Region: 3/24/2017 |
| Spain | Secretaria i Coordinació Comitè Ètic d'Investigació Clínica del Hospital Germans Trias i Pujol  Carretera Del Canyet S/n,  Badalona, 08916  Spain | F77-ES10002  HOSP. PROVINCIAL DE PONTEVEDRA  Calle Simon Bolivar, s/n Dermatology, 3th floor  Pontevedra, Spain, 36003  Spain  Received drug | Region: 3/24/2017 |
| Spain | Secretaria i Coordinació Comitè Ètic d'Investigació Clínica del Hospital Germans Trias i Pujol  Carretera Del Canyet S/n,  Badalona, 08916  Spain | F77-ES10003  HOSP. UNIV. 12 DE OCTUBRE  Centro de Actividades Ambulatorias,  Dermatology department, 2º floor, bloque B  Avenida de Córdoba s/n,  Madrid, Spain, 28041  Spain  Received drug | Region: 3/24/2017 |
| Spain | Secretaria i Coordinació Comitè Ètic d'Investigació Clínica del Hospital Germans Trias i Pujol  Carretera Del Canyet S/n,  Badalona, 08916  Spain | F77-ES10004  HOSP. UNIV. LA PAZ  Paseo de la Castellana, 261, UCICEC, Maternity Building, 2nd floor  Madrid, Spain, 28046  Spain  Received drug | Region: 3/24/2017 |
| Spain | Secretaria i Coordinació Comitè Ètic d'Investigació Clínica del Hospital Germans Trias i Pujol  Carretera Del Canyet S/n,  Badalona, 08916  Spain | F77-ES10006  HOSP. GRAL. UNIV. DE ALICANTE  Av. Pintor Baeza, 12 6th fllor, CCEE Building  Alicante, Spain, 03010  Spain  Received drug | Region: 3/24/2017 |
| Spain | Secretaria i Coordinació Comitè Ètic d'Investigació Clínica del Hospital Germans Trias i Pujol  Carretera Del Canyet S/n,  Badalona, 08916  Spain | F77-ES10007  HOSP. UNIV. I POLITECNI LA FE  Fernando Abril Martorell nº 106,  Dermatology department, Tower C 7th floor,  Valencia, Spain, 046026  Spain  Received drug | Region: 3/24/2017 |
| Spain | Secretaria i Coordinació Comitè Ètic d'Investigació Clínica del Hospital Germans Trias i Pujol  Carretera Del Canyet S/n,  Badalona, 08916  Spain | F77-ES10008  HOSP. UNIV. GERMANS TRIAS I PUJOL  Ctra De Canyet S/N, Dermatology, Ground Floor  Badalona, Spain, 08916  Spain  Received drug | Region: 3/24/2017 |
| Spain | Secretaria i Coordinació Comitè Ètic d'Investigació Clínica del Hospital Germans Trias i Pujol  Carretera Del Canyet S/n,  Badalona, 08916  Spain | F77-ES10009  HOSP. UNIV. INFANTA LEONOR  Gran Vía del Este, 80,Dermatology Department  Madrid, Spain, 28031  Spain  Received drug | Region: 3/24/2017 |
| Spain | Secretaria i Coordinació Comitè Ètic d'Investigació Clínica del Hospital Germans Trias i Pujol  Carretera Del Canyet S/n,  Badalona, 08916  Spain | F77-ES10010  HOSP. UNIV. DE BASURTO  Calle Montevideo Nº 18,Dermatology Department, Arrupe Pavilion 3rd floor  Bilbao Vizcaya, Spain, 48009  Spain  Received drug | Region: 3/24/2017 |
| Spain | Secretaria i Coordinació Comitè Ètic d'Investigació Clínica del Hospital Germans Trias i Pujol  Carretera Del Canyet S/n,  Badalona, 08916  Spain | F77-ES10012  HOSP. UNIV. FUNDACION ALCORCON  C/ Budapest, 1, Dermatology Deparment  Alcorcon, Spain, 28922  Spain  Received drug | Region: 3/24/2017 |
| Spain | Secretaria i Coordinació Comitè Ètic d'Investigació Clínica del Hospital Germans Trias i Pujol  Carretera Del Canyet S/n,  Badalona, 08916  Spain | F77-ES10013  HOSP. DE MANISES  Av. Generalitat Valenciana 50,  Dermatology Department  1st floor  Manises, Spain, 46940  Spain  Received drug | Region: 3/24/2017 |
| Spain | Secretaria i Coordinació Comitè Ètic d'Investigació Clínica del Hospital Germans Trias i Pujol  Carretera Del Canyet S/n,  Badalona, 08916  Spain | F77-ES10014  HOSP. DE LA SANTA CREU I SANT PAU  c/ Mas Casanovas 90,Bloque A Planta (-2) Centro Agdac  Barcelona, Spain, 8041  Spain  Received drug | Region: 3/24/2017 |
| Spain | Secretaria i Coordinació Comitè Ètic d'Investigació Clínica del Hospital Germans Trias i Pujol  Carretera Del Canyet S/n,  Badalona, 08916  Spain | F77-ES10015  HOSP. DEL MAR  Passeig Maritim de la Barceloneta 25-29, Dermatology Department  Barcelona, Spain, 08003  Spain  Received drug | Region: 3/24/2017 |
| Spain | Secretaria i Coordinació Comitè Ètic d'Investigació Clínica del Hospital Germans Trias i Pujol  Carretera Del Canyet S/n,  Badalona, 08916  Spain | F77-ES10016  HOSP. REINA SOFIA  (Hosp. Provincial) Avenida Menendez Pidal S/n,Dermatology 1st floor  Cordoba, Spain, 14004  Spain  Received drug | Region: 3/24/2017 |
| United States Of America | Sterling IRB  6300 Powers Ferry Road, Suite 600-351,  Atlanta, GA, 30339  United States Of America | F77-US10002  Academic Dermatology Associates  1203 Coal Avenue SE,  Albuquerque, NM, 87106  United States Of America  Received drug | Site, Central: 6/1/2017 |
| United States Of America | Sterling IRB  6300 Powers Ferry Road, Suite 600-351,  Atlanta, GA, 30339  United States Of America | F77-US10004  Somerset Skin Centre  255 Kirts Blvd, Suite 100,  Troy, MI, 48084  United States Of America  Received drug | Site, Central: 5/19/2017 |
| United States Of America | Sterling IRB  6300 Powers Ferry Road, Suite 600-351,  Atlanta, GA, 30339  United States Of America | F77-US10005  Clinical Partners  1524 Atwood Avenue, Suite 330,  Johnston, RI, 02919  United States Of America  Received drug | Site, Central: 4/18/2017 |
| United States Of America | Sterling IRB  6300 Powers Ferry Road, Suite 600-351,  Atlanta, GA, 30339  United States Of America | F77-US10007  Oregon Dermatology and Research Center  2565 NW Lovejoy, Suite 200,  Portland, OR, 97210  United States Of America  Received drug | Site, Central: 3/31/2017 |
| United States Of America | Sterling IRB  6300 Powers Ferry Road, Suite 600-351,  Atlanta, GA, 30339  United States Of America | F77-US10008  Advanced Medical Research  5730 Glenridge Drive, Suite T-100,  Sandy Spring, GA, 30328  United States Of America  Received drug | Site, Central: 5/23/2017 |
| United States Of America | Sterling IRB  6300 Powers Ferry Road, Suite 600-351,  Atlanta, GA, 30339  United States Of America | F77-US10012  Virginia Clinical Research  400 Gresham Dr,  Norfolk, VA, 23322  United States Of America  Received drug | Site, Central: 5/30/2017 |
| United States Of America | Henry Ford Health System IRB  2799 West Grand Blvd, CFP Basement 046, N/A,  Detroit, MI, 48202  United States Of America | F77-US10013  Henry Ford Medical Center  3031 West Grand Blvd. Ste 800,  Detroit, MI, 49202  United States Of America  Received drug | Site, Local: 4/14/2017 |
| United States Of America | Sterling IRB  6300 Powers Ferry Road, Suite 600-351,  Atlanta, GA, 30339  United States Of America | F77-US10014  Hamzavi Dermatology  2950 Keewahdin Road,  Fort Gratiot, MI, 48059  United States Of America  Received drug | Site, Central: 5/9/2017 |
| United States Of America | Sterling IRB  6300 Powers Ferry Road, Suite 600-351,  Atlanta, GA, 30339  United States Of America | F77-US10015  Indiana Clinical Trial Center  824 Edwards Drive, Suite 172,  Plainfield, IN, 46168  United States Of America  Received drug | Site, Central: 4/20/2017 |
| United States Of America | Sterling IRB  6300 Powers Ferry Road, Suite 600-351,  Atlanta, GA, 30339  United States Of America | F77-US10016  Modern Research Associates  9101 N. Central Expressway, Suite 170,  Dallas, TX, 75231  United States Of America  Received drug | Site, Central: 5/5/2017 |
| United States Of America | Sterling IRB  6300 Powers Ferry Road, Suite 600-351,  Atlanta, GA, 30339  United States Of America | F77-US10017  Southern California Dermatology  1125 E 17th St,  Santa Ana, CA, 92701  United States Of America  Received drug | Site, Central: 5/31/2017 |
| United States Of America | Sterling IRB  6300 Powers Ferry Road, Suite 600-351,  Atlanta, GA, 30339  United States Of America | F77-US10019  Minnesota Clinical Study Center  7205 University Avenue NE,  Fridley, MN, 55432  United States Of America  Received drug | Site, Central: 5/12/2017 |
| United States Of America | Kaiser Permanente Southern California IRB  393 E Walnut St Fl 2,  Pasadena, CA, 91188  United States Of America | F77-US10021  Southern California Permanente Medical Group  4867 W Sunset Blvd, Department of Dermatology,  Los Angeles, CA, 90027  United States Of America  Received drug | Site, Local: 4/19/2017 |
| United States Of America | Sterling IRB  6300 Powers Ferry Road, Suite 600-351,  Atlanta, GA, 30339  United States Of America | F77-US10022  Dermatology Associates of Seattle  1730 Minor Avenue Suite 1000,  Seattle, WA, 98101-1498  United States Of America  Received drug | Site, Central: 5/10/2017 |
| United States Of America | Western Institutional Review Board  1019 39th Avenue SE, Suite 120,  Puyallup, WA, 98374  United States Of America | F77-US10023  The Ohio State University  540 Officenter Pl,  Gahanna, OH, 43230  United States Of America  Received drug | Site, Local: 6/15/17 |
| United States Of America | Sterling IRB  6300 Powers Ferry Road, Suite 600-351,  Atlanta, GA, 30339  United States Of America | F77-US10024  Arlington Dermatology  5301 Keystone Ct.,  Rolling Meadows, IL, 60008  United States Of America  Received drug | Site, Central: 3/14/2017 |
| United States Of America | Sterling IRB  6300 Powers Ferry Road, Suite 600-351,  Atlanta, GA, 30339  United States Of America | F77-US10025  Oregon Medical Research Center  9495 SW Locust Street, Suite G,  Portland, OR, 97223  United States Of America  Received drug | Site, Central: 4/12/2017 |
| United States Of America | Sterling IRB  6300 Powers Ferry Road, Suite 600-351,  Atlanta, GA, 30339  United States Of America | F77-US10026  Renstar Medical Research  21 NE 1st Ave,  Ocala, FL, 34470  United States Of America  Received drug | Site, Central: 2/17/2017 |
| United States Of America | Sterling IRB  6300 Powers Ferry Road, Suite 600-351,  Atlanta, GA, 30339  United States Of America | F77-US10027  MedDerm Associates  501 Washington Street, Suite 502,  San Diego, CA, 92103  United States Of America  Received drug | Site, Central: 4/26/2017 |
| United States Of America | Sterling IRB  6300 Powers Ferry Road, Suite 600-351,  Atlanta, GA, 30339  United States Of America | F77-US10028  DermAssociates, PC  15245 Shady Grove Road, Suite 480,  Rockville, MD, 20850  United States Of America  Received drug | Site, Central: 4/14/2017 |
| United States Of America | Sterling IRB  6300 Powers Ferry Road, Suite 600-351,  Atlanta, GA, 30339  United States Of America | F77-US10030  Atlanta Dermatology, Vein & Research Center  11800 Atlantis Place,  Alpharetta, GA, 30022  United States Of America  Received drug | Site, Central: 4/20/2017 |
| United States Of America | Sterling IRB  6300 Powers Ferry Road, Suite 600-351,  Atlanta, GA, 30339  United States Of America | F77-US10031  Dermatology Consulting Services, PLLC  2444 N Main St,  High Point, NC, 27262  United States Of America  Received drug | Site, Central: 6/5/2017 |
| United States Of America | Sterling IRB  6300 Powers Ferry Road, Suite 600-351,  Atlanta, GA, 30339  United States Of America | F77-US10033  Menter Dermatology Research Institute  3900 Junius St,  Dallas, TX, 75246-1615  United States Of America  Received drug | Site, Central: 5/30/2017 |
| United States Of America | Sterling IRB  6300 Powers Ferry Road, Suite 600-351,  Atlanta, GA, 30339  United States Of America | F77-US10034  University of Pittsburgh Department of Dermatology  3601 5th Ave, Falk Building, 5th Floor,  Pittsburgh, PA, 15213  United States Of America  Received drug | Site, Central: 5/24/2017 |
| United States Of America | Western Institutional Review Board  1019 39th Avenue SE, Suite 120,  Puyallup, WA, 98374  United States Of America | F77-US10035  University of Alabama Birmingham  Dermatology at the Whitaker Clinic, John N. Whitaker Building, 500 22nd Street South, Suite 3400  Birmingham, AL, 35233  United States Of America  Received drug | Site, Local: 6/28/2017 |
| United States Of America | Sterling IRB  6300 Powers Ferry Road, Suite 600-351,  Atlanta, GA, 30339  United States Of America | F77-US10036  Dermatology Clinical Research Center of San Antonio  7810 Louis Pasteur Dr Ste 200,  San Antonio, TX, 78229  United States Of America  Received drug | Site, Central: 2/14/2017 |
| United States Of America | Sterling IRB  6300 Powers Ferry Road, Suite 600-351,  Atlanta, GA, 30339  United States Of America | F77-US10037  Olympian Clinical Research  4238 W. Kennedy Blvd.,  Tampa, FL, 33609  United States Of America  Received drug | Site, Central: 5/26/2017 |
| United States Of America | Sterling IRB  6300 Powers Ferry Road, Suite 600-351,  Atlanta, GA, 30339  United States Of America | F77-US10038  Windsor Dermatology  59 One Mile Rd Ext Ste G,  East Windsor, NJ, 08520-2505  United States Of America  Received drug | Site, Central: 5/1/2017 |
| United States Of America | Sterling IRB  6300 Powers Ferry Road, Suite 600-351,  Atlanta, GA, 30339  United States Of America | F77-US10039  Suzanne Bruce and Associates - The Center for Skin Research  1900 Saint James Pl, Ste 650,  Houston, TX, 77056-4132  United States Of America  Received drug | Site, Central: 5/15/2017 |
| United States Of America | Sterling IRB  6300 Powers Ferry Road, Suite 600-351,  Atlanta, GA, 30339  United States Of America | F77-US10040  Central Sooner Research  900 N Porter Avenue, Suite 207,  Norman, OK, 73071  United States Of America  Received drug | Site, Central: 4/20/2017 |
| United States Of America | Sterling IRB  6300 Powers Ferry Road, Suite 600-351,  Atlanta, GA, 30339  United States Of America | F77-US10041  Marietta Dermatology Clinical Research  111 Marble Mill Road,  Marietta, GA, 30060  United States Of America  Received drug | Site, Central: 3/16/2017 |
| United States Of America | Sterling IRB  6300 Powers Ferry Road, Suite 600-351,  Atlanta, GA, 30339  United States Of America | F77-US10042  Dermatologists of Greater Columbus  2359 E. Main St,  Bexley, OH, 43209  United States Of America  Received drug | Site, Central: 5/5/2017 |
| United States Of America | Sterling IRB  6300 Powers Ferry Road, Suite 600-351,  Atlanta, GA, 30339  United States Of America | F77-US10043  San Luis Dermatology & Laser Clinic, Inc  15 Santa Rosa St,  San Luis Obispo, CA, 93405  United States Of America  Received drug | Site, Central: 5/5/2017 |
| United States Of America | Sterling IRB  6300 Powers Ferry Road, Suite 600-351,  Atlanta, GA, 30339  United States Of America | F77-US10044  Dermatology Specialists  3629 Vista Way,  Oceanside, CA, 92056  United States Of America  Received drug | Site, Central: 5/19/2017 |
| United States Of America | Sterling IRB  6300 Powers Ferry Road, Suite 600-351,  Atlanta, GA, 30339  United States Of America | F77-US10045  Park Avenue Dermatology  906 Park Avenue,  Orange Park, FL, 32073  United States Of America  Received drug | Site, Central: 4/25/2017 |
| United States Of America | Sterling IRB  6300 Powers Ferry Road, Suite 600-351,  Atlanta, GA, 30339  United States Of America | F77-US10047  Austin Dermatology Associates  3705 Medical Parkway, Suite 340,  Austin, TX, 78705  United States Of America  Received drug | Site, Central: 5/5/2017 |
| United States Of America | Sterling IRB  6300 Powers Ferry Road, Suite 600-351,  Atlanta, GA, 30339  United States Of America | F77-US10050  Great Lakes Research Group  200 S. Wenona # 170,  Bay City, MI, 48706  United States Of America  Received drug | Site, Central: 5/30/2017 |
| United States Of America | Sterling IRB  6300 Powers Ferry Road, Suite 600-351,  Atlanta, GA, 30339  United States Of America | F77-US10051  Clinical Research Center of Connecticut  27 Hospital Avenue; Suite 206,  Danbury, CT, 06810  United States Of America  Received drug | Site, Central: 5/30/2017 |
